# Interactions Between Cadmium Contamination and Mineral Sorbents in Shaping Heavy Metal Bioaccumulation in Soil and Plant Biomass

**DOI:** 10.3390/molecules31203784

**Published:** 2026-10-21

**Authors:** Jadwiga Wyszkowska, Agata Borowik, Magdalena Zaborowska, Jan Kucharski

**Affiliations:** Department of Soil Science and Microbiology, Faculty of Agriculture and Forestry, University of Warmia and Mazury in Olsztyn, Plac Łódzki 3, 10-727 Olsztyn, Poland; agata.borowik@uwm.edu.pl (A.B.); m.zaborowska@uwm.edu.pl (M.Z.); jan.kucharski@uwm.edu.pl (J.K.)

**Keywords:** heavy metals, translocation, bioaccumulation, metal uptake, remediation, *Zea mays*, sorbents

## Abstract

By disrupting soil ecosystem functioning, cadmium threatens food safety. Therefore, a significant research imperative is to develop effective remediation strategies and understand the relationships between cadmium content in soil and plants and the content of other metals. This study aimed to evaluate the effects of calcium carbonate, sepiolite, expanded clay, zeolite, and molecular sieve on the growth and physiological response of *Zea mays* grown in cadmium-contaminated soil. Plant growth and development were assessed together with the translocation and bioaccumulation of cadmium and other metals (Pb, Cr, Cu, Zn, Fe, Ni, and Mn) and changes in soil physicochemical properties. The study was conducted as a pot experiment using uncontaminated soil and soil contaminated with 15 and 30 mg Cd kg^−1^. Depending on the contamination level, cadmium reduced the leaf greenness index of *Zea mays* by 10–16%, the yield of the aboveground parts by 60–76%, and root yield by 60–69%. The tested sorbents modified these responses mainly by altering the soil physicochemical properties. The molecular sieve was the most effective, reducing the negative effect of cadmium on the yield of the aboveground parts and roots by an average of 31% while not significantly affecting the leaf greenness index. Cadmium increased the translocation factor of all analyzed elements except cadmium itself, indicating its potential to affect the homeostasis of other elements in the plant. Heavy metal bioaccumulation in the roots of *Zea mays* was significantly greater than in the aboveground parts. Cadmium altered the bioaccumulation of individual elements to different extents, but these effects could be modified through the appropriate selection of mineral sorbents. These findings make an important contribution to the development of effective methods for remediating cadmium-contaminated soils.

## 1. Introduction

Cadmium (Cd), due to its high mobility [1], toxicity [2], and ability to accumulate in plants [3,4], is among the most problematic soil contaminants [5], making it one of the key risk factors affecting the stability of soil ecosystems [6]. A meta-analysis conducted by Hou et al. [7] showed that the rate of exceedance of permissible Cd concentrations in soils is the highest among all heavy metals, reaching approximately 9.0%. These data underscore the key importance of cadmium in global soil quality monitoring systems. The highest risk has been reported in northern and central India, Pakistan, Bangladesh, southern China, southern Thailand, and Cambodia, as well as in Iran, Turkey, Ethiopia, Nigeria, South Africa, Mexico, and Cuba [7]. In the European Union, soil cadmium concentrations also increased by approximately 50% during the 20th century [8]. In regions with a long history of industrialization, such as the United States and Europe, long-term industrial and mining activities have resulted in numerous contaminated areas where Cd accumulation is widespread [9]. A spatial analysis based on more than 21,000 soil samples collected across the European Union (EU) and the United Kingdom showed that cadmium concentrations in topsoil range from 0.07 mg kg^−1^ to 65.4 mg kg^−1^ [8].

In Europe, Directive (EU) 2025/2360 of the European Parliament and of the Council on soil monitoring and resilience (Soil Monitoring Law, SML) has further reinforced soil quality monitoring [10]. It pays particular attention to inorganic contaminants, including heavy metals, which disrupt soil ecosystem functioning and threaten food safety [7,11]. The scale of cadmium contamination in soils [4,12], together with SML requirements, highlights the need not only for precise monitoring of contaminated areas [7,8], but also for the implementation of effective remediation strategies for cadmium-contaminated soils [13,14].

Heavy metals are difficult to remove from degraded areas [15] because remediation must both reduce contamination levels and use technologies tailored to the specific physicochemical properties of the site [16,17]. In recent years, stabilizing materials such as mineral sorbents have received particular attention because they can reduce heavy metal mobility [18,19]. Their application enables the long-term binding of cadmium within the soil matrix, thereby reducing its bioavailability and decreasing the risk of its transfer to plants and other environmental compartments [20].

Among these materials, molecular sieve, expanded clay, zeolite, sepiolite, and calcium carbonate deserve particular attention [21,22]. Owing to its precisely defined pore structure, molecular sieve enables the selective sorption of metal ions. Expanded clay, as a material characterized by high porosity and a large specific surface area, can reduce Cd mobility through physical and chemical adsorption. The mechanisms underlying Cd immobilization by expanded clay share certain similarities with those of biochar, which may involve physical adsorption, electrostatic interactions, ion exchange, surface complexation, and the precipitation of sparingly soluble metal compounds [23,24]. Zeolite, owing to its high cation exchange capacity and stable silicate structure, effectively immobilizes Cd through cation exchange and surface complexation [22,25]. Sepiolite, characterized by its channel-like structure and high surface reactivity, has the capacity to bind Cd through surface adsorption and complexation [26]. Calcium carbonate, in turn, contributes to Cd immobilization by increasing soil pH and promoting the formation of sparingly soluble metal forms [21].

Despite numerous studies demonstrating the effectiveness of these sorbents for remediating cadmium-contaminated soils, comprehensive studies examining how sorbent properties interact with plant responses to influence the bioaccumulation of cadmium and other heavy metals remain scarce. Previous studies have focused mainly on individual sorbents, while studies comparing mineral sorbents with different physicochemical properties with respect to the uptake of other metals and changes in Cd transport and distribution within plant biomass are much less common. Wang et al. [27] and Motrescu et al. [28] report that these processes depend strongly on sorbent physicochemical properties, nutrient availability, and microbial activity in the rhizosphere.

These issues are particularly important when biomass from contaminated areas is considered for use, because potentially high heavy metal contents may pose a risk to human and animal health [29,30]. Nevertheless, consistent with the principles of the circular economy and the RED III Directive [31], such biomass can be used for bioenergy production [32,33], particularly because its calorific value is comparable to that of biomass from uncontaminated areas [34,35]. This makes it an attractive energy feedstock given the growing global demand for primary energy, approximately 10% of which is currently supplied by biomass [36].

The present study addresses a research gap arising from limited knowledge of how different mineral sorbents modify cadmium behavior in soil under complex chemical interactions and how these processes affect the accumulation of other metals in *Zea mays*. Understanding these relationships is essential for developing effective and sustainable remediation strategies consistent with the requirements of the SML Directive, which aims to improve soil health and resilience. This study focuses on the interactions between cadmium-contaminated soil and mineral sorbents and their effects on the bioaccumulation of trace elements in soil and plant biomass. In the present study, *Zea mays* was used as a test plant to assess metal uptake and accumulation, whereas bioenergy production was considered as a potential application of biomass from contaminated soils. The investigation addresses a gap in current knowledge regarding how mineral sorbents with diverse physicochemical properties modify cadmium behavior in soils and how these processes affect the uptake, bioaccumulation, and translocation of cadmium and other metals in *Zea mays*. Understanding these relationships is essential for developing effective and sustainable remediation strategies in line with the objectives of the SML Directive, which aims to improve soil health and resilience.

The study aimed to assess the effects of calcium carbonate, sepiolite, expanded clay, zeolite, and molecular sieve on the response of *Zea mays* grown in cadmium-contaminated soil, with particular emphasis on changes in plant growth, the leaf greenness index (SPAD), metal uptake, metal translocation and bioaccumulation, and modifications of the chemical and physicochemical properties of soil that influence the bioavailability of these elements. Research in this area provides valuable insights into metal immobilization and translocation, supporting the development of effective methods for the remediation of cadmium-contaminated soils. The authors formulated the following research hypotheses: (i) cadmium contamination of soil reduces *Zea mays* biomass and leaf greenness index (SPAD) values and alters the uptake, bioaccumulation, and translocation of other metals, (ii) mineral sorbents reduce the mobility and bioavailability of cadmium and other metals, (iii) mineral sorbents limit the translocation of heavy metals to the aboveground parts of plants, thereby increasing the phytostabilization potential of *Zea mays*, and (iv) sorbents characterized by high sorption capacity and the ability to increase soil pH exhibit the greatest effectiveness.

## 2. Results

### 2.1. Chemical and Physicochemical Properties of Cadmium-Contaminated Soil

Cadmium contamination of soil did not affect the contents of total carbon and total nitrogen (Table A1). These parameters changed only after sorbent application. Their addition increased the C_org_ content by an average of 2–10% and the N_total_ content by 3–13%, except for zeolite, which had the opposite effect in soil contaminated with 15 and 30 mg Cd kg^−1^. Soil reaction, expressed as pH, did not depend on the cadmium contamination level but was significantly modified by the sorbents. All sorbents, except expanded clay, increased soil pH. Zeolite increased soil pH from 4.46 to 4.60, while sepiolite, molecular sieve, and calcium carbonate increased it to 5.91, 6.23, and 6.23, respectively. Cadmium decreased the hydrolytic acidity of soil contaminated with 30 mg Cd kg^−1^, whereas the sorbents affected this parameter to varying degrees. Expanded clay and zeolite increased hydrolytic acidity by an average of 10% and 6%, respectively, whereas calcium carbonate and molecular sieve reduced it by 46% and sepiolite by 42%. The sum of exchangeable base cations decreased in cadmium-contaminated soil. Most sorbents increased the value of this parameter: calcium carbonate by an average of 21%, molecular sieve by 19%, zeolite by 3%, and sepiolite by 2%. Expanded clay had no effect on this property. The cation exchange capacity of soil, similarly to the sum of exchangeable base cations, was reduced by excess cadmium. The sorbents modified this parameter to varying degrees: calcium carbonate increased it by an average of 7%, molecular sieve by 5%, and zeolite by 4%. Expanded clay caused no changes, whereas sepiolite reduced the cation exchange capacity by 7%. The degree of soil saturation with base cations was not consistently related to the cadmium contamination level. Cadmium significantly reduced this parameter at 15 mg Cd kg^−1^, whereas at 30 mg Cd kg^−1^ it caused no significant change. In contrast, some sorbents, such as calcium carbonate and molecular sieve, increased the degree of saturation by an average of 13%, while sepiolite increased it by 10%. The remaining sorbents negatively affected this property.

An elevated level of cadmium in soil without added sorbents contributed to a decrease in lead and chromium contents, regardless of the cadmium dose applied (Table A2). A decrease in copper, nickel, and manganese contents was observed only at a dose of 30 mg Cd kg^−1^. In addition, cadmium increased zinc and iron contents.

The effects of sorbents on Cd and Cu contents in uncontaminated soil were neutral, whereas their effects on the remaining elements varied. Sepiolite reduced Pb, Cr, and Fe contents, expanded clay reduced Cr and Fe contents, molecular sieve reduced Pb content, and calcium carbonate reduced Cr content. All sorbents increased Zn and Mn contents, except calcium carbonate. In addition, zeolite increased Cr and Ni contents, expanded clay increased Ni content, and molecular sieve increased Fe content. In cadmium-contaminated soil, none of the sorbents significantly changed the Cd content, whereas their effects on the contents of the other analyzed metals varied.

In soil contaminated with 15 mg and 30 mg Cd kg^−1^, individual sorbents increased the contents of the following metals: calcium carbonate—Pb, Cr, and Mn, sepiolite—Cr and Mn, expanded clay—Pb, Cu, Zn, and Mn, zeolite—Cr, Cu, Ni, and Mn, and molecular sieve—Cu and Ni. Thus, the sorbents did not affect the analyzed heavy metals in the same way, regardless of the level of cadmium contamination. Some of them increased the contents of specific elements only in soil with a higher level of cadmium contamination, namely: calcium carbonate—Ni, sepiolite—Pb and Zn, expanded clay—Ni, zeolite—Pb, and molecular sieve—Pb and Mn.

### 2.2. Phytotoxic Effects of Cadmium on Zea mays

**Figure 1:**
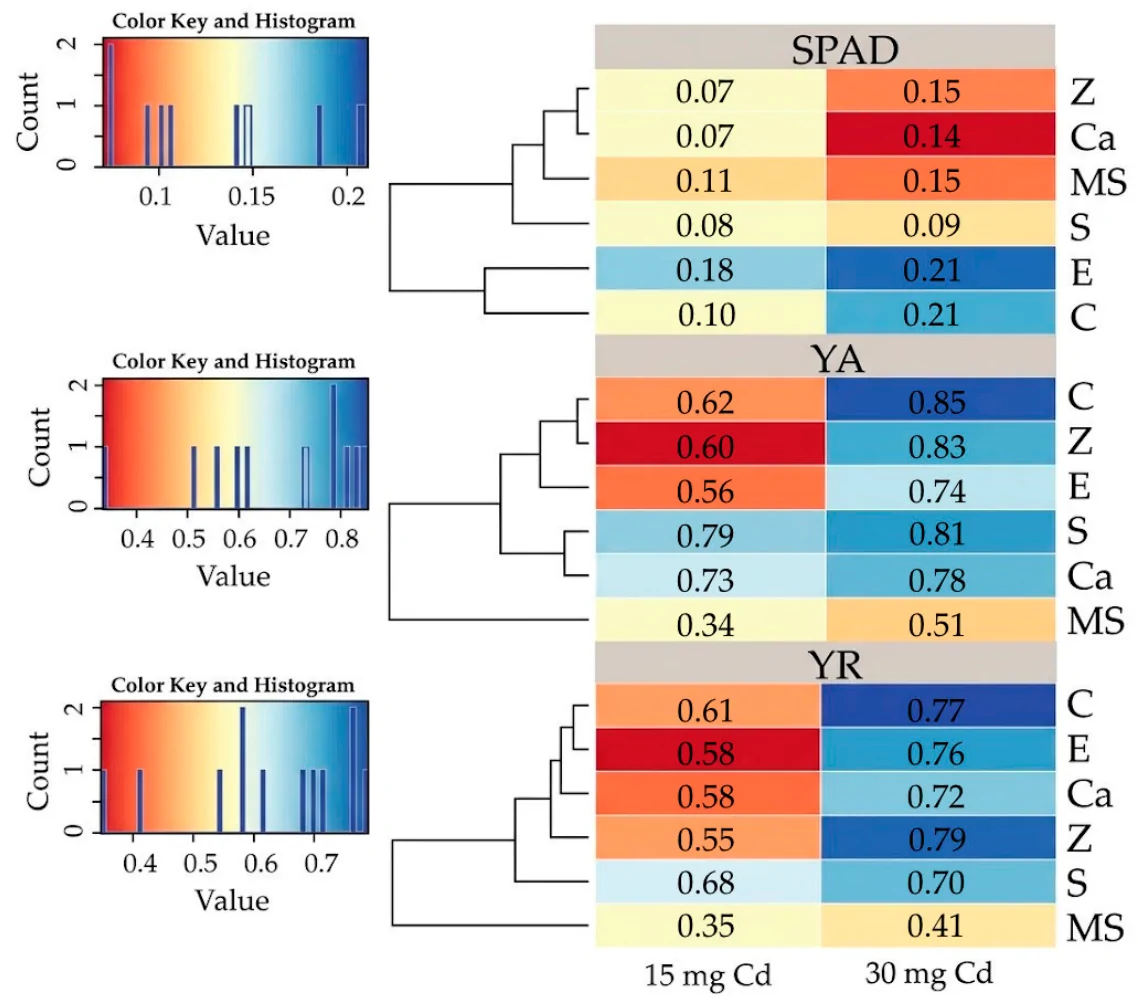
Toxic effect (TE) of cadmium on *Zea mays*. Explanations: YA—yield of the aboveground parts of *Zea mays*; YR—root yield of *Zea mays*; C—control; E—expanded clay; Ca—calcium carbonate; Z—zeolite; S—sepiolite; MS—molecular sieve; SPAD—leaf greenness index.

Cadmium contamination of soil negatively affected both the leaf greenness index (SPAD) of *Zea mays* and the biomass of the aboveground parts and roots (Figure 1; Figure S1). This element had a more destructive effect on biomass than on the leaf greenness index. The toxic effect index for SPAD ranged, on average, from 0.102 to 0.158, for the yield of the aboveground parts, from 0.603 to 0.755, and for root yield, from 0.599 to 0.691. The first, lower value resulted from the lower cadmium contamination level (15 mg Cd kg^−1^), whereas the second, higher value corresponded to the higher contamination level (30 mg Cd kg^−1^).

The toxicity index (TE) of cadmium for the analyzed traits in treatments containing sorbents was partially masked by the effect of some sorbents on SPAD values and the yield of the aboveground parts (Figure S1). Calcium carbonate significantly reduced the leaf greenness index of *Zea mays* grown in uncontaminated soil. The same sorbent, similar to sepiolite, also reduced the aboveground biomass. Nevertheless, all sorbents, except expanded clay, reduced the negative effect of cadmium on SPAD, while expanded clay, zeolite, and molecular sieve also mitigated its effect on the aboveground biomass. In addition, calcium carbonate and molecular sieve reduced the cadmium toxicity index for roots. Overall, molecular sieve reduced cadmium toxicity to *Zea mays* to the greatest extent. This sorbent decreased the mean toxicity index for SPAD from 0.154 to 0.126, for the aboveground biomass from 0.735 to 0.424, and for root biomass from 0.690 to 0.380.

### 2.3. Trace Elements in Zea mays and Their Translocation

#### 2.3.1. Trace Element Content in *Zea mays*

The addition of sorbents to soil uncontaminated with cadmium reduced the contents of cadmium, chromium, zinc, and nickel in all sorbent treatments, copper in treatments with calcium carbonate, sepiolite, and molecular sieve, iron following the application of sepiolite and zeolite, and manganese following the application of molecular sieve. In contrast, calcium carbonate, zeolite, and molecular sieve increased the lead content, expanded clay and molecular sieve increased the iron content, zeolite increased the copper content, while calcium carbonate, sepiolite, and zeolite increased the manganese content.

In cadmium-contaminated soil, individual sorbents exerted consistent effects only on the contents of selected elements, with both decreases and increases in their contents in the aboveground parts of *Zea mays* grown in soil containing 15 mg Cd kg^−1^ and 30 mg Cd kg^−1^ being considered significant. Thus, molecular sieve reduced the contents of cadmium, zinc, and manganese, expanded clay reduced the contents of chromium and nickel, calcium carbonate reduced the contents of chromium, zinc, nickel, and manganese, and sepiolite reduced the contents of chromium, copper, zinc, nickel, and manganese. In contrast, expanded clay increased the contents of cadmium, zinc, and manganese, zeolite increased the cadmium content, and molecular sieve increased the copper content.

In the roots of *Zea mays* (Table A3) grown in soil uncontaminated with cadmium and without sorbent addition, the following element contents were determined (mg kg^−1^ d.m.): Cd—0.05, Pb—2.00, Cr—14.80, Cu—3.70, Zn—51.40, Ni—9.95, Fe—2472.55, and Mn—153.40. Cadmium contamination of soil increased the contents of Cd and Cu in the roots and decreased the contents of the remaining elements analyzed.

The sorbents applied to cadmium-contaminated soil significantly reduced the contents of cadmium, lead, zinc, nickel, and manganese in the roots of *Zea mays*, regardless of sorbent type. Their effects on the remaining elements varied. For example, all sorbents except molecular sieve reduced chromium content, but this effect was observed only in plants grown in soil contaminated with 15 mg Cd kg^−1^. At the higher cadmium contamination level, the sorbents did not affect chromium content. All sorbents except zeolite reduced copper content in roots of plants grown in soil with the higher cadmium contamination level. In contrast, molecular sieve increased copper content regardless of the contamination level. Iron content in roots depended on both the level of cadmium contamination and the type of sorbent applied. In plants grown in soil contaminated with 15 mg Cd kg^−1^, all sorbents reduced iron content. In contrast, in the roots of *Zea mays* grown in soil containing 30 mg Cd kg^−1^, expanded clay, zeolite, and molecular sieve increased iron content.

#### 2.3.2. Translocation of Trace Elements in *Zea mays*

**Figure 2:**
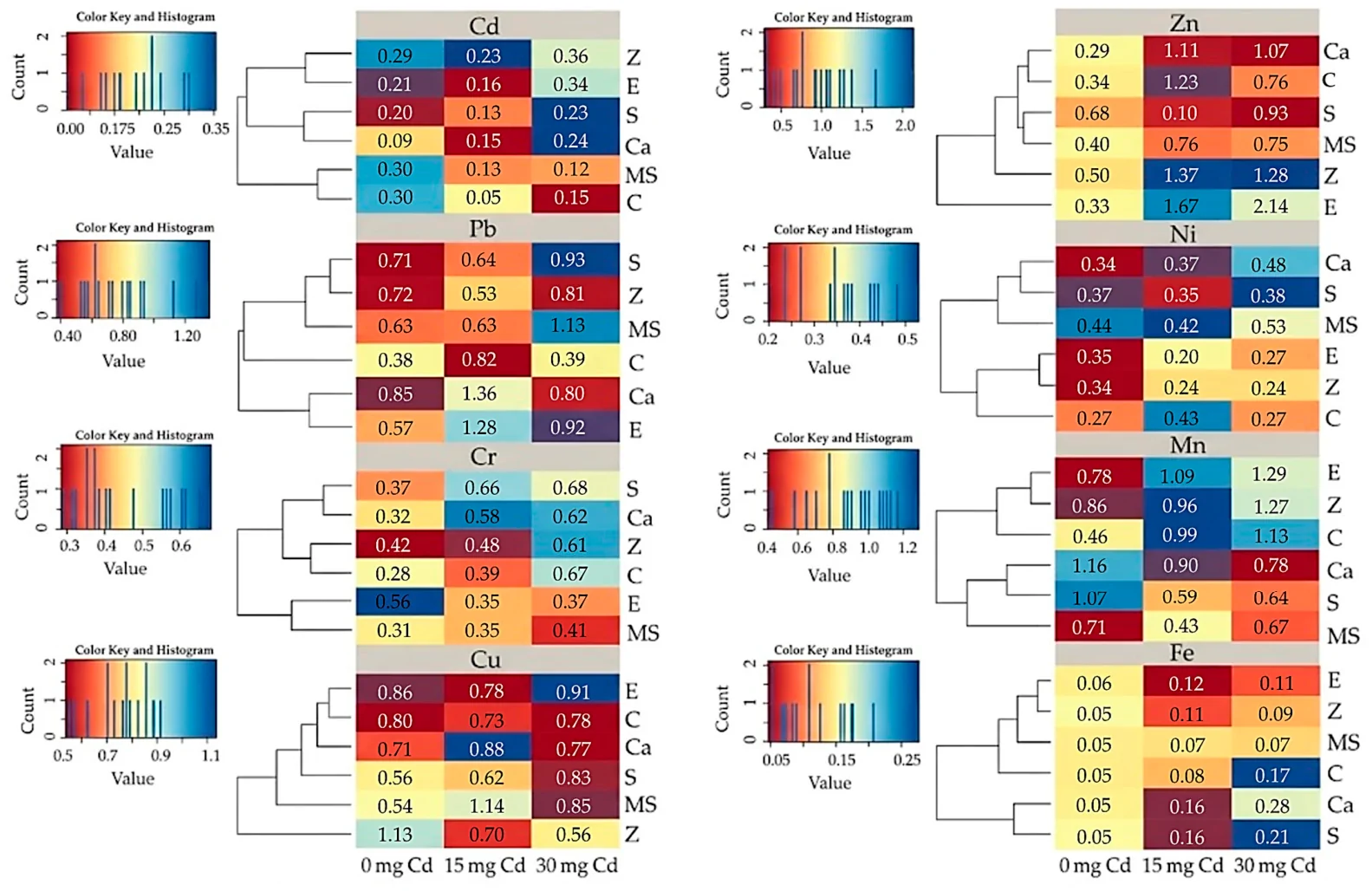
Translocation factor (TF) of trace elements in *Zea mays.* Explanations: C—control; E—expanded clay; Ca—calcium carbonate; Z—zeolite; S—sepiolite; MS—molecular sieve.

In *Zea mays* grown in soil uncontaminated with cadmium, the translocation factors of individual heavy metals were as follows: cadmium—0.300, lead—0.375, chromium—0.284, copper—0.797, zinc—0.339, nickel—0.271, iron—0.054, and manganese—0.460 (Figure 2). Cadmium contamination of soil altered the values of this index, although the direction of these changes often depended on the contamination level. In plants grown in control soil without sorbent addition, increasing soil cadmium concentrations reduced the translocation factor for cadmium and copper, while they increased the translocation of lead, chromium, zinc, iron, and manganese at both levels of cadmium contamination.

Changes in the translocation factor (TF) of the elements resulted not only from the presence of cadmium in the soil. The applied sorbents also had a significant effect. In *Zea mays* grown in uncontaminated soil, calcium carbonate and sepiolite reduced the TF values for cadmium and copper, expanded clay reduced the TF for cadmium, while molecular sieve reduced the TF for copper. All sorbents increased the translocation of lead, chromium, nickel, and manganese, zeolite additionally increased the translocation of copper and zinc, while sepiolite and molecular sieve increased the translocation of zinc. None of the sorbents affected the TF value for iron.

Cadmium contamination of soil partially modified the effects of sorbents on element translocation. All sorbents increased lead translocation in *Zea mays* grown in soil contaminated with 30 mg Cd kg^−1^. A similar effect was observed for cadmium and zinc translocation, except for the molecular sieve. Sepiolite, expanded clay, and molecular sieve increased copper translocation, calcium carbonate, sepiolite, and molecular sieve increased nickel translocation, calcium carbonate and sepiolite increased iron translocation, and expanded clay and zeolite increased manganese translocation. In contrast, calcium carbonate, expanded clay, zeolite, and molecular sieve reduced chromium translocation, zeolite reduced copper and nickel translocation, expanded clay, zeolite, and molecular sieve reduced iron translocation, and calcium carbonate, sepiolite, and molecular sieve reduced manganese translocation.

### 2.4. Uptake and Accumulation of Trace Elements in Zea mays

#### 2.4.1. Uptake of Trace Elements by *Zea mays*

Uptake of individual chemical elements from soil by *Zea mays* decreased progressively as cadmium contamination increased (Table S1). Cadmium was the only exception, with uptake increasing proportionally to its soil content and by 2204% after contamination with 30 mg Cd kg^−1^. In contrast, uptake of the remaining metals decreased by 80% for lead, 83% for chromium, 63% for copper, 69% for zinc, 71% for nickel, 82% for iron, and 66% for manganese at this contamination level.

The effects of sorbents on elemental uptake by *Zea mays* varied depending on the metal analyzed. In fact, only nickel uptake was reduced by all sorbents, with the greatest reductions recorded for sepiolite (52%) and calcium carbonate (49%) and the smallest for molecular sieve (19%).

Calcium carbonate and sepiolite reduced the uptake of all metals analyzed by *Zea mays*. Calcium carbonate reduced uptake by 10% for Pb, 42% for Cd, and 49% for Ni, whereas sepiolite reduced it by 40% for Cd and 53% for Zn. Expanded clay significantly reduced lead uptake by 22%, chromium uptake by 38%, and nickel uptake by 34%, while increasing cadmium uptake by 17%, zinc uptake by 15%, and manganese uptake by 11%. Zeolite reduced chromium uptake by 23%, zinc uptake by 18%, nickel uptake by 26%, and manganese uptake by 6%, while increasing cadmium uptake by 15% and iron uptake by 17%. The molecular sieve reduced cadmium uptake by 65%, nickel uptake by 19%, and manganese uptake by 58%, while increasing lead uptake by 38%, chromium uptake by 16%, copper uptake by 89%, zinc uptake by 8%, and iron uptake by 27%.

#### 2.4.2. Bioaccumulation of Trace Elements in *Zea mays*

**Figure 3:**
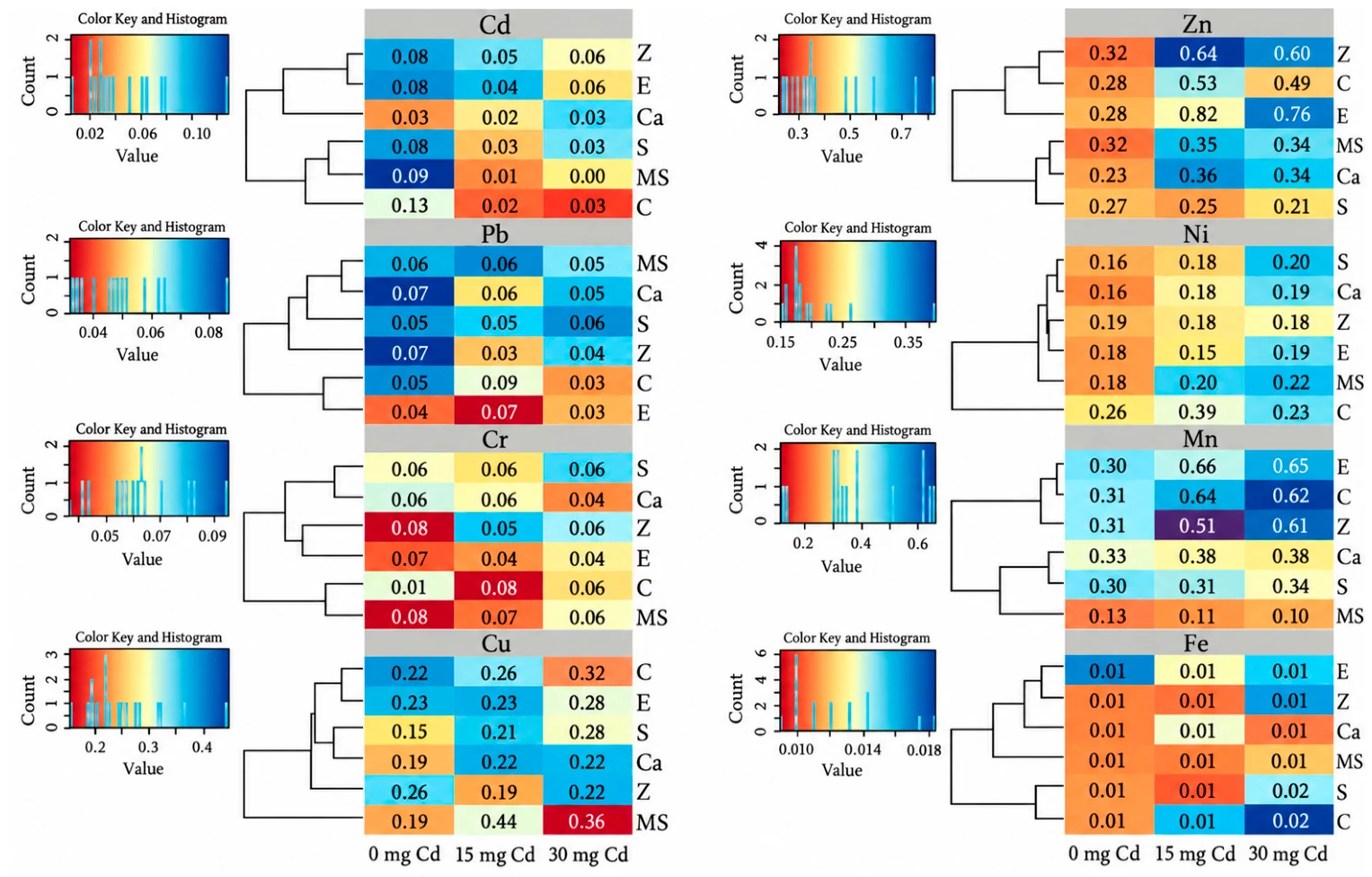
Heavy metal bioaccumulation factor in the aboveground parts of *Zea mays* (BAF_A_). Explanations: C—control; E—expanded clay; Ca—calcium carbonate; Z—zeolite; S—sepiolite; MS—molecular sieve.

The bioaccumulation factor of metals in the aboveground parts of *Zea mays* was low. Mean values, irrespective of the level of soil contamination with cadmium and the type of sorbent applied, ranged from 0.013 for iron to 0.443 for manganese (Figure 3). The bioaccumulation of the remaining elements showed intermediate values between these extremes. The applied sorbents reduced the bioaccumulation of chromium, copper, zinc, and nickel, regardless of sorbent type. However, they did not affect iron bioaccumulation. Calcium carbonate also reduced the value of this factor for cadmium and manganese, sepiolite and molecular sieve reduced it for cadmium, expanded clay, zeolite, and molecular sieve reduced it for lead, whereas calcium carbonate, sepiolite, and zeolite reduced it for manganese.

**Figure 4:**
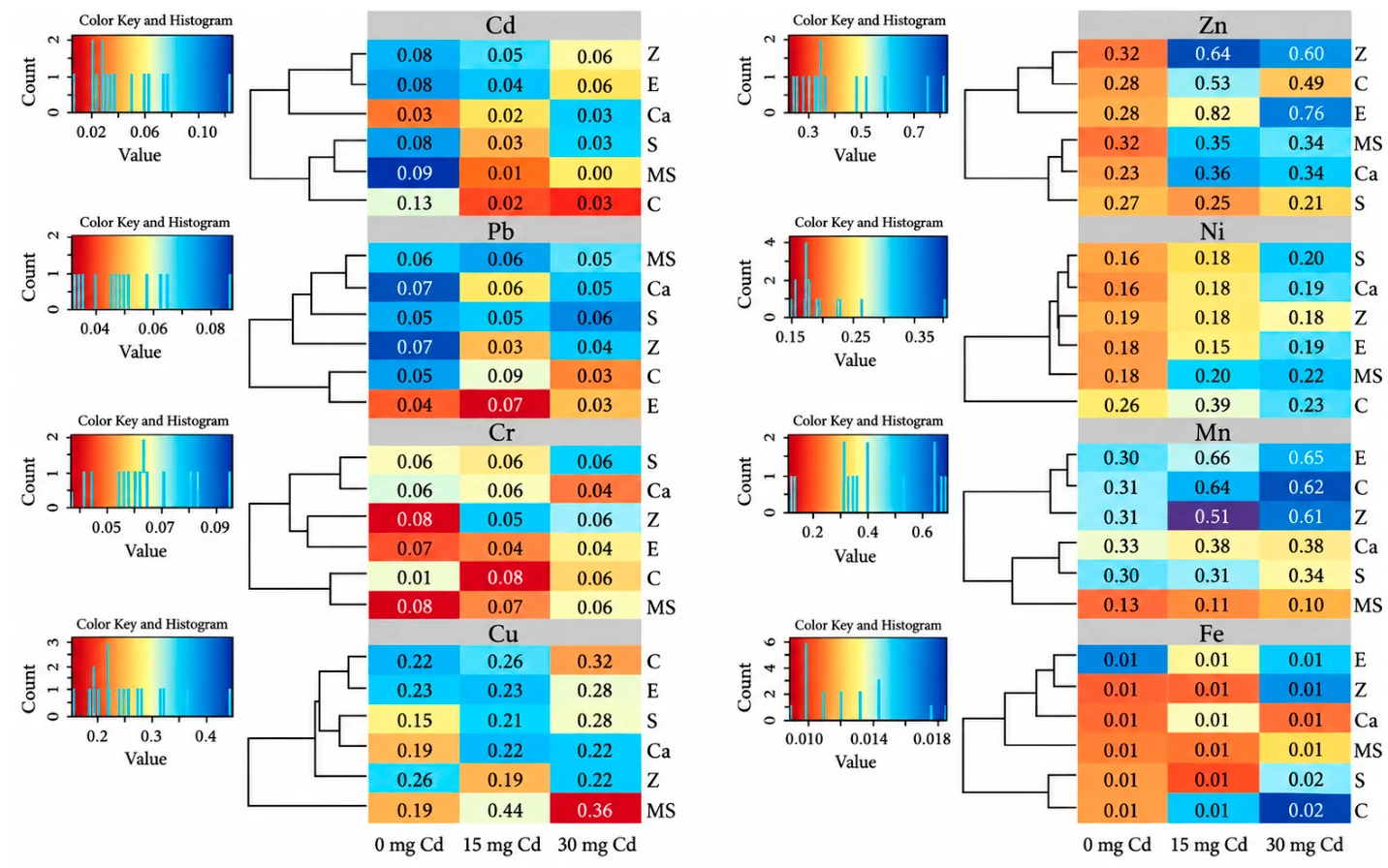
Heavy metal bioaccumulation factor in the roots of *Zea mays* (BAF_R_). Explanations: C—control; E—expanded clay; Ca—calcium carbonate; Z—zeolite; S—sepiolite; MS—molecular sieve.

The bioaccumulation of heavy metals in the roots of *Zea mays* was significantly higher than in the aboveground parts (Figure 4). An elevated cadmium content in soil decreased the bioaccumulation of cadmium, lead, chromium, zinc, and iron in the roots, while increasing the bioaccumulation of copper, nickel, and manganese. All applied sorbents reduced the bioaccumulation of the analyzed metals, with the exception of molecular sieve for copper and zeolite and molecular sieve for iron.

### 2.5. Relationships Between Zea mays Yield and the Uptake and Translocation of Trace Elements

**Figure 5:**
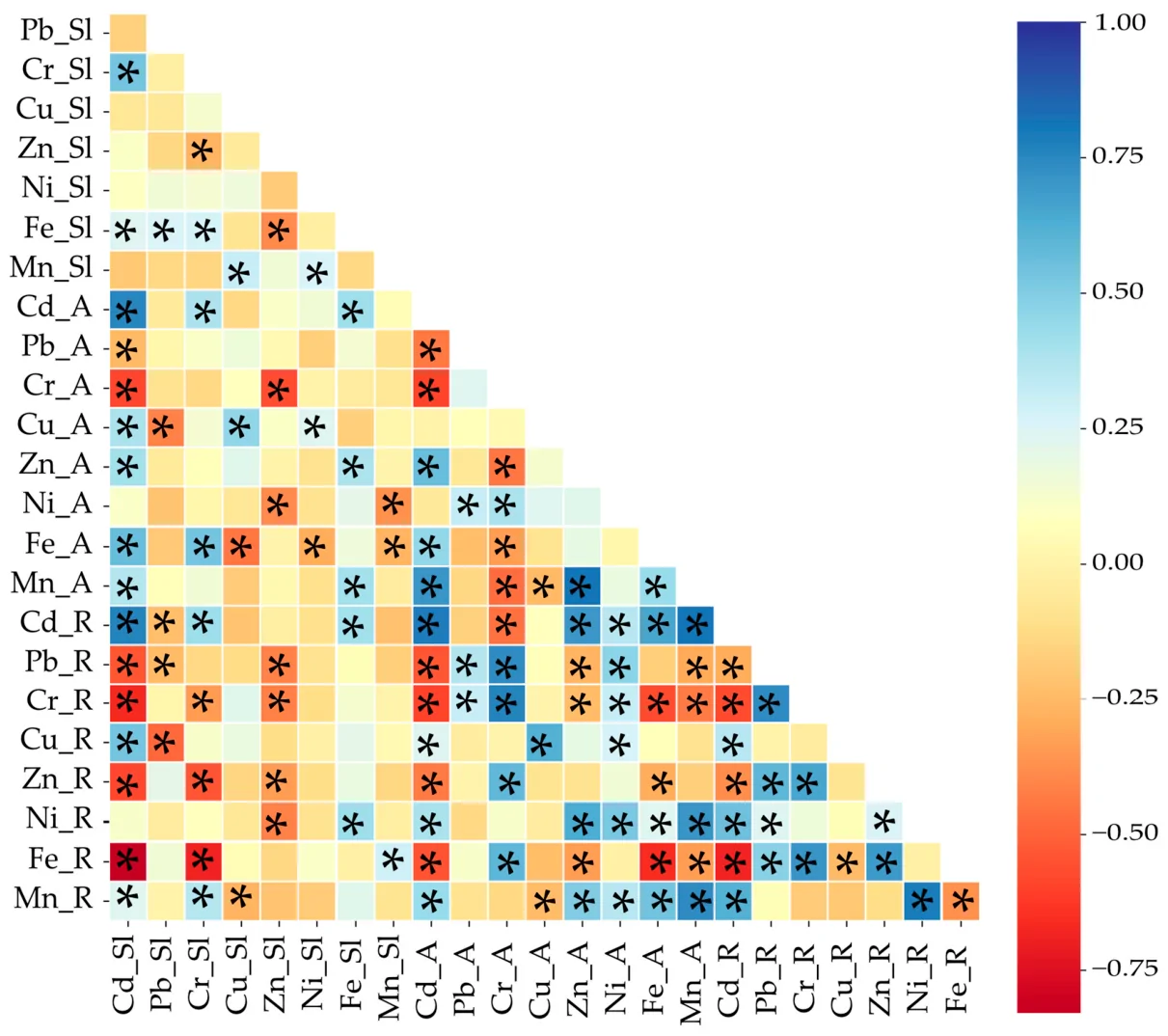
Pearson’s correlation coefficients between heavy metal contents in soil (Sl), the aboveground parts (A), and roots (R) of *Zea mays*. Asterisks (*) indicate statistically significant correlation coefficients at *p* < 0.05; *n* = 72.

The cadmium content in soil was positively correlated with the contents of cadmium, copper, zinc, iron, and manganese in the aboveground parts and with the contents of cadmium and copper in the roots, as well as with the concentrations of chromium and iron in the soil itself (Figure 5). Negative correlations were recorded between the cadmium content in soil and the contents of lead and chromium in both plant organs, as well as the contents of zinc and iron in the roots.

The cadmium content in the aboveground parts of *Zea mays* was positively correlated with the contents of zinc, iron, and manganese in these organs and with the contents of cadmium, nickel, and manganese in the roots. Negative correlations were found between the cadmium content in the aboveground parts and the contents of lead and chromium in both the aboveground parts and roots, as well as the contents of zinc and iron in the roots. A significant positive correlation was observed between the cadmium content in the roots and the contents of manganese, nickel, and copper in these organs, whereas negative correlations were found with the contents of iron, zinc, chromium, and lead.

**Figure 6:**
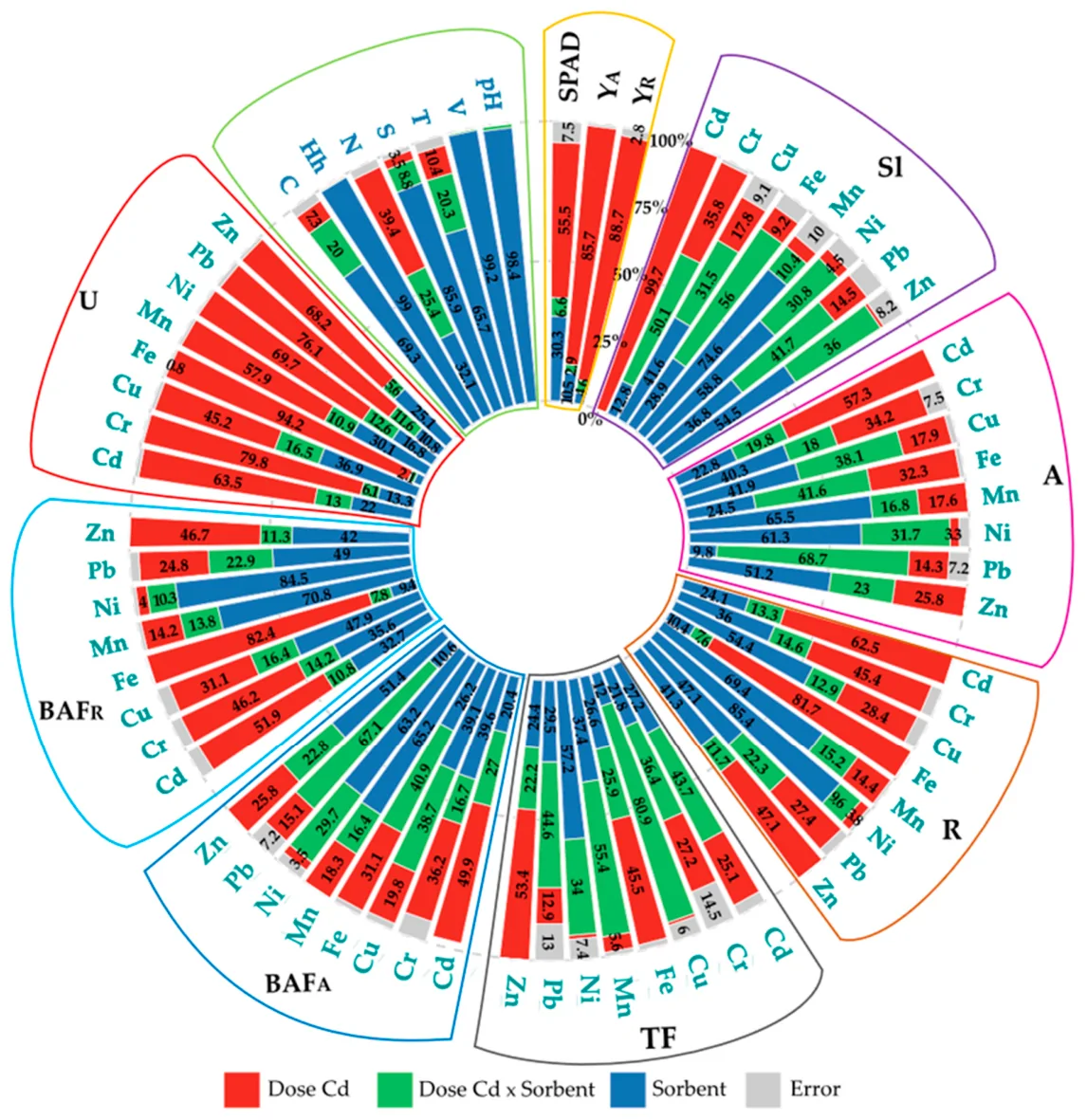
Effect sizes of cadmium (Cd) and mineral amendments (M) on 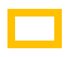 the leaf greenness index (SPAD), yield of the aboveground parts (YA) and roots (YR) of *Zea mays*, 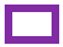 heavy metal contents in soil (Sl), 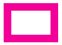 heavy metal contents in the aboveground parts (A), 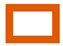 heavy metal contents in the roots (R), 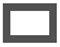 heavy metal translocation factor (TF), 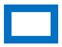 heavy metal bioaccumulation factor in the aboveground parts of *Zea mays* (BAF_A_), 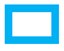 heavy metal bioaccumulation factor in the roots of *Zea mays* (BAF_R_), 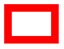 heavy metal uptake (U) by *Zea mays*, and 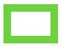 the chemical and physicochemical properties of soil, expressed as eta squared (%).

Overall, the results clearly indicate that cadmium contamination of the soil had the greatest effect on *Zea mays* yield, the leaf greenness index (SPAD), and the uptake of heavy metals from soil (Figure 6). Cadmium exerted a strong effect on the bioaccumulation of iron, cadmium, chromium, zinc, and copper in the roots, whereas its effect on metal bioaccumulation in the aboveground parts was considerably weaker. Similarly, cadmium had a stronger effect on the contents of heavy metals in the roots than in the aboveground parts (Table A4).

The effect of the applied sorbents on the growth and development of *Zea mays* and on metal uptake from soil was clearly smaller than the effect caused by cadmium addition. Calcium carbonate reduced Cd uptake by *Zea mays* by 42%, sepiolite by 40%, and molecular sieve by 65%, whereas expanded clay and zeolite increased Cd uptake by 17% and 15%, respectively (Table S1). Sorbents that reduced Cd uptake also decreased its bioaccumulation factor (BAF) and translocation from roots to aboveground tissues.

The sorbents had the greatest effect on the physicochemical properties of soil and on the soil contents of C_org_, manganese, nickel, zinc, copper, lead, and iron. They also exerted a significant effect on the bioaccumulation of nickel and manganese in the aboveground parts and roots, as well as on the contents of these elements in plant biomass.

To facilitate the comparison of individual mineral sorbents, their contrasting effects on Cd uptake by *Zea mays* are additionally illustrated in Figure S2, highlighting the particularly high effectiveness of molecular sieve.

## 3. Discussion

In the present study, cadmium contamination of soil significantly reduced the yield of the aboveground biomass and roots of *Zea mays*. At a cadmium contamination level of 30 mg Cd kg^−1^ soil d.m., the toxicity index was 0.755 for the yield of the aboveground parts and 0.691 for root yield. The significant reduction in root biomass observed in our study is probably related to the root system being the first site of plant contact with cadmium present in soil [37,38]. High Cd accumulation in roots disrupts cell division, inhibits root elongation, damages cell membranes, and alters cell wall structure, thereby reducing water and nutrient uptake [39]. Yue et al. [40] and Zhang et al. [41] emphasized that cadmium-induced stress in maize roots leads to disturbances in the expression of genes associated with growth regulation, auxin transport, and root system functioning. The reduction in the aboveground biomass of plants may, in turn, result from cadmium-induced disturbances in photosynthetic processes [41,42]. Cadmium enhances the formation of reactive oxygen species, which damage cell membranes and chloroplast structures [43,44]. It induces lipid peroxidation and cell damage, alters enzyme activity, causes protein degradation, and induces DNA damage and mutations [45]. Haider et al. [37] reported that this metal inhibits chlorophyll synthesis, damages the photosynthetic apparatus, and reduces the rates of photosynthesis and transpiration, as well as water-use efficiency in maize [43,46], thereby reducing plant growth.

Cadmium also significantly reduced the leaf greenness index (SPAD) of *Zea mays*, which is considered one of the most sensitive and earliest indicators of cadmium phytotoxicity in maize [47]. A decrease in the SPAD index indicates progressive degradation of photosynthetic pigments under the influence of cadmium [37]. This is consistent with the results of our previous studies [3,34] and those of other authors [37,48], which demonstrated a significant relationship between the efficiency of the photosynthetic apparatus and *Zea mays* biomass. Because cadmium persists in soil, reducing its bioavailability and uptake by plants is particularly important for food safety [7,48]. Therefore, research on the use of mineral sorption materials for cadmium stabilization in the soil environment is particularly relevant [18,34]. In the present study, molecular sieve showed the highest effectiveness among all sorbents analyzed, significantly limiting the negative effects of cadmium on both the leaf greenness index and the aboveground and root biomass of *Zea mays*. This effect may be attributed to the high porosity, large specific surface area, and cation exchange capacity of the molecular sieve, which enable effective immobilization of heavy metals [49]. In our study, in addition to the molecular sieve, the other mineral sorbents, except expanded clay, also reduced the negative effect of cadmium on SPAD, while sepiolite and zeolite also mitigated its effect on aboveground biomass. The positive effect of zeolite may result from its high cation exchange capacity and ability to immobilize cadmium [50], whereas the effect of sepiolite may be associated with its capacity for adsorption and complexation of this metal [18,51]. Calcium carbonate reduced Cd toxicity to roots, which was associated with increased soil pH and a potential reduction in Cd availability for plant uptake [52,53]. Elevated soil pH may decrease Cd solubility by facilitating its adsorption onto mineral surfaces and promoting the formation of sparingly soluble Cd compounds under suitable environmental conditions. Changes in the surface charge of soil constituents may further enhance this process by promoting the retention of Cd^2+^ ions at higher pH levels [54].

The results clearly demonstrate that cadmium contamination of soil increases the accumulation of this element in the aboveground parts and roots of *Zea mays*, reflecting its transfer from soil to plant tissues [55,56]. The increased contents of Cd, Zn, Fe, and Mn in the aboveground parts of maize may indicate alterations in plant ion homeostasis. This phenomenon may result from competition between Cd and certain divalent metals, such as Ca^2+^, Fe^2+^, Mn^2+^, and Zn^2+^, for common membrane transporters, including IRT1 (iron-regulated transporter 1), ZIP (ZRT/IRT-like protein), and NRAMP (natural resistance-associated macrophage protein) [57,58]. An additional factor increasing cadmium uptake is its ability to enter cells through calcium channels, which may increase cadmium content, particularly within the root system [4].

In the present study, increasing soil contamination with cadmium disrupted the uptake of trace elements by *Zea mays*. Excess cadmium caused a significant increase in Cd uptake, accompanied by a decrease in the uptake of Pb, Cr, Cu, Zn, Ni, Fe, and Mn. Elik and Gül [38] and Gao et al. [45] indicated that excess Cd disrupts metal homeostasis, which may lead to reduced uptake of other trace elements and impaired root system functioning. These relationships are consistent with the mechanisms of ionic competition and membrane transport described above. Cadmium ions may interact with various cellular components and disrupt normal cell functioning [56,59]. A key process contributing to Cd accumulation in aboveground plant tissues is its translocation from roots via the xylem [60].

The translocation factor (TF) was used to assess the transfer of trace elements from roots to aboveground tissues of *Zea mays*, while the bioaccumulation factor (BAF) was used to evaluate their accumulation in plant tissues relative to their concentrations in soil. Both indices provide valuable information on trace element transfer within the soil–plant system and the potential risk of their entry into the food chain [61,62,63]. The results indicate that increasing Cd content in soil significantly altered the translocation and bioaccumulation of heavy metals in *Zea mays*, with the direction of the response depending on both the element and the sorbent applied. Heavy metal bioaccumulation in the roots of *Zea mays* was significantly higher than in the aboveground parts, confirming that roots constitute the main barrier limiting the translocation of trace elements to aboveground organs. In roots, metal ions can be rapidly bound by phytochelatins and metallothioneins, thereby partially limiting their translocation to the aboveground parts [61,64].

In plants grown in soil without sorbent addition, higher cadmium content in soil reduced the translocation factor of cadmium and copper while increasing the translocation of lead, chromium, zinc, iron, and manganese. Similar relationships were reported by Shan et al. [61], who found low TF values for Cd (0.04–0.12) in maize grown in soil contaminated with this element. Wang et al. [65] and Elik and Gül [45] demonstrated that excess cadmium in soil modifies the uptake and distribution not only of cadmium but also of other elements in *Zea mays,* which may result from competition among metal ions for the same transport systems and from disturbances in plant mineral homeostasis. The decrease in the bioaccumulation of Cd, Pb, Cr, Zn, and Fe in roots, together with increased accumulation of Cu, Ni, and Mn observed in our study, may result from competitive interactions among metal ions during uptake by membrane transporters [45,66]. Dawar et al. [67] indicated that Cd often competes with Fe, Zn, and Ca for common transport systems and may stimulate Mn and Ni uptake through compensatory mechanisms associated with oxidative stress.

Particularly noteworthy are the results concerning the effects of mineral sorbents on heavy metal contents in *Zea mays*, as all sorbents applied to cadmium-contaminated soil significantly reduced the contents of cadmium, lead, zinc, nickel, and manganese in the roots of *Zea mays*. This indicates effective stabilization of heavy metals in the rhizosphere [68,69]. Consistent with our findings, Akbar et al. [70] showed that mineral and organic amendments reduced the bioavailability of Cd and Pb, leading to a significant decrease in their contents in maize roots. In the aboveground parts, however, we found that molecular sieve reduced cadmium, zinc, and manganese contents, expanded clay reduced chromium and nickel, calcium carbonate reduced chromium, zinc, nickel, and manganese, and sepiolite reduced chromium, copper, zinc, nickel, and manganese. In contrast, expanded clay increased the contents of cadmium, zinc, and manganese, zeolite increased cadmium, and molecular sieve increased copper. The variable effectiveness of individual sorbents indicates that immobilization mechanisms are selective and depend both on sorbent properties and on ionic competition among metals [18,53,69]. Shahkolaie et al. [69] and Akbar et al. [70] indicate that the variable effectiveness of sorbents observed in the present study may result from their selective interactions with individual heavy metals. Increased immobilization of one trace element may lead to increased mobility or bioavailability of other metal ions.

We demonstrated that sepiolite and calcium carbonate were particularly effective in reducing the uptake of all analyzed metals. Sepiolite reduced the uptake of individual elements by as much as 40–53%. This is supported by the findings of Wang et al. [18], who demonstrated that the application of sepiolite and its composites reduced the content of bioavailable Cd in soil by up to 36.8% and significantly reduced metal accumulation in maize grain. This effect may be associated with the large specific surface area of sepiolite, its cation exchange capacity, and its ability to adsorb and immobilize heavy metal ions [71]. Calcium carbonate was effective in reducing metal uptake because it modified soil reaction, which plays an important role in determining heavy metal bioavailability. In our study, calcium carbonate increased soil pH from 4.36 to 6.23. An increase in soil pH leads to the precipitation of cadmium as sparingly soluble compounds and increases the sorption of metal cations by the soil sorption complex [72]. The remaining sorbents showed more selective effects. Expanded clay reduced the uptake of Pb, Cr, and Ni while increasing the uptake of Cd, Zn, and Mn, whereas zeolite reduced the uptake of Cr, Zn, Ni, and Mn and increased the accumulation of Cd and Fe. Molecular sieve was the most effective sorbent in reducing Cd concentrations in both roots and aboveground tissues of *Zea mays*. It also reduced Ni and Mn concentrations but increased those of Pb, Cr, Cu, Zn, and Fe. This suggests that their effects on element bioavailability depend on specific sorption properties and changes in the ionic balance of the soil [18,38,45,73].

In the present study, the mineral sorbents significantly affected the translocation and bioaccumulation of trace elements in *Zea mays*. In general, they increased the translocation factors (TF) for Pb, Cd, and Zn, whereas their effects on the remaining elements varied depending on sorbent type, with TF values for Cr, Fe, and Mn translocation most often reduced. Chen et al. [51] demonstrated that sepiolite effectively stabilized Cd and Pb, thereby reducing their accumulation in maize. Similar immobilization properties of sepiolite-containing composites were reported by Wang et al. [18], who observed a 29–37% reduction in bioavailable Cd and a decrease in its content in maize grain of up to 45%.

Both the present study and previous reports [18,71,74] indicate that the effectiveness of sorbents in limiting the transfer of heavy metals to the aboveground plant parts depends not only on their capacity to adsorb metal ions but also on their effects on the physicochemical properties of soil. In the present study, mineral sorbents significantly modified the chemical properties of the soil, with calcium carbonate and molecular sieve showing the most favorable effects. These sorbents increased soil pH, the sum of exchangeable base cations, cation exchange capacity, and base saturation while simultaneously reducing hydrolytic acidity. Sepiolite also improved most of the properties analyzed, whereas zeolite and expanded clay exerted weaker and more variable effects. The application of mineral amendments at a rate of 20 g kg^−1^ soil did not significantly increase the total metal concentrations in soil. These relationships are consistent with earlier reports by Tian et al. [71] and Bondar et al. [75] indicating that mineral sorbents characterized by a high cation exchange capacity promote the accumulation of base cations in the soil sorption complex and the immobilization of cadmium. These findings demonstrate that calcium carbonate and molecular sieve exerted particularly beneficial effects on soil chemical properties, potentially reducing Cd availability for plant uptake. In summary, consideration of the interactions between cadmium contamination of soil and the application of mineral sorbents in shaping heavy metal bioaccumulation in soil and plant biomass indicates that *Zea mays* is an effective phytostabilizing plant and that sepiolite, calcium carbonate, and molecular sieve are among the most effective materials for reducing the bioavailability and accumulation of heavy metals. These findings are consistent with current knowledge on heavy metal stabilization in soils and reduced metal uptake by *Zea mays*, confirming the effectiveness of these sorbents in limiting the environmental risk associated with cadmium contamination [11,18,59,61,76].

## 4. Materials and Methods

### 4.1. Pot Experiment Design

The experiment was conducted during the summer of 2022, from early June to late July, i.e., during months characterized by relatively stable thermal conditions favorable for plant growth. The present study is exploratory and provides a foundation for the design of future field experiments. In 2022, the mean annual temperature in Olsztyn was 8.9 °C, reflecting the typical climatic conditions of northeastern Poland. In June 2022, the mean air temperature was 17.9 °C, with minimum and maximum temperatures of 11.9 °C and 24.1 °C, respectively. Similar values were recorded in July, with a mean temperature of 18.0 °C and a range of 13.2–23.5 °C [77]. The experiment was conducted in a completely randomized design in the vegetation hall of the University of Warmia and Mazury in Olsztyn (northeastern Poland), with four replicates.

### 4.2. The Experimental Procedure

1. Selection and collection of soil from the arable layer, and soil characterization:
  - location: northeastern Poland, 53°45′36′′ N, 20°27′15′′ E;
  - particle size distribution: sandy loam (SL): sand (0.05–2 mm)—63.61%, silt (0.002–0.05 mm)—32.68%, clay (<0.002 mm)—3.71%.
2. Preparation of soil for the pot experiment:
  - air-drying the soil to constant weight;
  - sieving the soil through a 5 mm mesh;
  - determination of basic physicochemical properties: pH in 1 mol KCl dm^−3^—4.40, pH in H_2_O dm^−3^—5.52, hydrolytic acidity—26.10 mM(+) kg^−1^ d.m. of soil, exchangeable base cations—63.60 mM(+) kg^−1^ d.m. of soil, cation exchange capacity—89.70 mM(+) kg^−1^ d.m. of soil;
  - determination of soil chemical properties: 10.00 g C_org_ kg^−1^ of soil dry matter (d.m.), 0.83 g N_total_ kg^−1^ of d.m., 0.115 mg Cd kg^−1^ of d.m., 1.659 mg Pb kg^−1^ of d.m., 4.409 mg Cr kg^−1^ of d.m., 1.315 mg Cu kg^−1^ of d.m., 5.356 mg Zn kg^−1^ of d.m., 1.019 mg Ni kg^−1^ of d.m., 1270.462 mg Fe kg^−1^ of d.m., 23.019 mg Mn kg^−1^ of d.m.
3. Weigh 3.4 kg d.m. of soil into polyethene pots measuring 16 cm in height and 17 cm in diameter.
4. Soil fertilization to meet the nutritional requirements of the test plant:
  - 150 mg N kg^−1^ soil as CO(NH_2_)_2_;
  - 50 mg P kg^−1^ soil as KH_2_PO_4_;
  - 150 mg K kg^−1^ soil as KH_2_PO_4_ + KCl;
  - 20 mg Mg kg^−1^ soil as MgSO_4_·7H_2_O.
5. Division of the soil into three series according to the level of contamination:
  - series 1: uncontaminated soil;
  - series 2: soil + 15 mg Cd kg^−1^ d.m. of soil;
  - series 3: soil + 30 mg Cd kg^−1^ d.m. of soil.
6. Application of mineral phytoremediation amendments to each experimental series:
  - control—without amendments;
  - CaCO_3_ at 1.96 g kg^−1^, corresponding to 1.5 times the hydrolytic acidity;
  - Bio.Zeo.S.01 zeolite at 20 g kg^−1^ d.m. of soil;
  - sepiolite at 20 g kg^−1^ d.m. of soil;
  - expanded clay at 20 g kg^−1^ d.m. of soil;
  - molecular sieve at 20 g kg^−1^ d.m. of soil.
7. Sowing of *Zea mays* (Zm), cultivar DS1897B.

A total of 72 pots were prepared, representing combinations of 6 sorbent treatments (including the control without sorbent), 3 levels of cadmium contamination, and 4 replicates. The soil in each pot was adjusted to 60% of its maximum water-holding capacity, and this moisture level was maintained throughout the 55-day experimental period. On the day the experiment was established, eight maize seeds (*Zea mays* L., cultivar DS1897B) were sown in each pot, and after emergence, four uniform plants were retained. When the plants reached BBCH stage 51 (beginning of tassel emergence), the leaf greenness index (SPAD) was measured on each leaf of each plant, and the values were averaged. Measurements were performed using a chlorophyll meter (Chlorophyll Meter, Spectrum Technologies, KONICA MINOLTA, Osaka, Japan). At the end of the experiment, the aboveground parts and roots were collected, and soil samples were obtained for subsequent chemical and physicochemical analyses under laboratory conditions. After drying, the plant material was ground to <1 mm.

### 4.3. Characteristics of the Materials Used

Cadmium was added to the soil as 3CdSO_4_·8H_2_O (Sigma-Aldrich, Saint Louis, MO, USA). Urea (CO(NH_2_)_2_) supplied nitrogen, potassium dihydrogen phosphate (KH_2_PO_4_) supplied phosphorus, potassium dihydrogen phosphate together with potassium chloride (KCl) supplied potassium, and magnesium sulfate heptahydrate (MgSO_4_·7H_2_O) supplied magnesium. CO(NH_2_)_2_, KH_2_PO_4_, KCl, and MgSO_4_·7H_2_O were obtained from STANLAB (Lublin, Poland). Cadmium and the mineral fertilizers were thoroughly mixed with the soil, which was then incubated for 5 days at 60% of maximum water-holding capacity (WHC). Maize (*Zea mays* L.) was used as the test plant. It is one of the most widely cultivated cereal crops worldwide [78,79] because it adapts well to diverse environmental conditions [80]. Maize also shows relatively high tolerance to cadmium in soil [41,81], making it a suitable model for studies of heavy metal mobility and accumulation [82]. The resulting biomass can also be effectively used in the energy sector, particularly in bioenergy technologies [83,84], which increases the practical relevance of the study.

Five mineral materials with different chemical properties were used in the experiment to modify the growth conditions of *Zea mays* under cadmium stress:

Molecular sieve (MS)—a synthetic Silosiv A3-type zeolite consisting of hydrated aluminosilicate with a uniform microporous structure and a pore diameter of approximately 0.3 nm. It is characterized by a slightly alkaline reaction (pH_KCl_ 8.5) and a low content of volatile substances, not exceeding 2.5% after ignition at 950 °C. Manufacturer: Sylosiv (Columbia, MD, USA).

Bio.Zeo.S.01 zeolite (Z)—a natural aluminosilicate of volcanic origin, composed predominantly of clinoptilolite (60%). The proportions of the major elements were as follows: Si—33.00%, Al—3.26%, K—1.17%, Ca—2.44%, Mg—0.56%, and Fe—0.52%. Manufacturer: BioDrain (Rzeszów, Poland).

Sepiolite (S)—a hydrated magnesium silicate with the formula Mg_4_[Si_6_O_15_(OH)_2_]·6H_2_O, characterized by a fibrous structure and pores with a diameter of approximately 1.4 nm—its pH_KCl_ is 7.1. Manufacturer: Sepiolsa Minersa Group (Guadalajara, Spain).

Expanded clay (E)—a lightweight ceramic aggregate produced by firing clay at approximately 1200 °C. The material is characterized by a broad particle-size range of 75–710 µm and a pH_KCl_ of 7.1. Manufacturer: GardenGURU (Wrocław, Poland).

Calcium carbonate (Ca)—finely crystalline CaCO_3_ in the form of a white powder with a molar mass of 100.09 g mol^−1^. Manufacturer: Chempur (Piekary Śląskie, Poland).

A detailed characterization of the mineral sorbents is provided in our previous studies [84,85].

### 4.4. Laboratory Analytical Methods

The aboveground parts and roots of maize plants were dried in a convection oven (Binder, D–78532, Tuttlingen, Germany) at 60 °C. Soil samples were air-dried under laboratory conditions at ambient temperature. Before the experiment, the particle-size distribution of the soil was determined, whereas the contents of organic carbon and total nitrogen, soil reaction, hydrolytic acidity, the sum of exchangeable base cations, and trace metals (Cd, Pb, Cr, Cu, Zn, Ni, Fe, and Mn) were determined before and after the experiment (Table 1). The same elements were also determined in the biomass of the aboveground parts and roots of maize after the experiment. Soil samples and plant material were subjected to microwave digestion using an UltraWAVE microwave digestion system (Milestone, Sorisole, Italy). For digestion, 5 cm^3^ of 65% HNO_3_ was added per 0.5 g of sample, and after completion of the digestion process, the solutions were made up to a final volume of 100 cm^3^ with demineralized water. The concentrations of the elements were determined by ICP–OES using a certified multielement standard solution (Certipur^®^, Merck, Darmstadt, Germany) containing 24 elements, including cadmium, lead, chromium, copper, zinc, nickel, iron, and manganese. Blank samples were also included in the analyses. All analyses were performed in four replicates.

The HAC and EBC values were used to calculate the soil cation exchange capacity (CEC), expressed in mmol(+) kg^−1^, as well as the base saturation (BS), expressed as a percentage.

### 4.5. Data Processing

Based on the dry matter yield of the aboveground parts and roots of maize grown in control and cadmium-contaminated soil, a set of indices was calculated to describe plant responses to heavy metal exposure. The analyses used the contents of cadmium, lead, chromium, copper, zinc, nickel, iron, and manganese determined in soil and in the biomass of the aboveground parts and roots. The study included calculations of the toxicity index, translocation factor, bioaccumulation factors for heavy metals in the aboveground parts and roots of *Zea mays*, and heavy metal uptake by the plants. These calculations were based on the equations presented in Table 2.

All data obtained during the study were subjected to statistical analysis using Statistica 13.3 [87], RStudio 2023.06.0 [88] with R 4.2.2 [89], and the gplots package 3.1.3 [90] (Table 3).

The analyses aimed to determine the effects of two experimental factors: the level of soil contamination with cadmium (factor I) and the type of sorbent applied (factor II) on the parameters studied. All statistical analyses were performed at significance levels of *p* = 0.05 and *p* = 0.01. The results were presented in tables as means ± SD, with values reported to three decimal places, and graphically using boxplots, heatmaps, and a circular stacked bar chart.

## 5. Conclusions

Cadmium contamination of soil significantly inhibited the growth and development of *Zea mays*, reducing the leaf greenness index and plant biomass. Maize showed phytostabilizing characteristics by accumulating most of the analyzed metals in the roots. The translocation factor differed among metals, with mean values ranging from 0.168 for Cd to 0.960 for Mn. Cadmium increased the bioaccumulation factor of manganese, nickel, and copper in both the roots and aboveground parts of the plants.

Mineral sorbents significantly reduced heavy metal bioaccumulation in the roots and aboveground parts of *Zea mays*. This effect is not always desirable for the remediation of cadmium-contaminated soils, although it may improve the quality of the plant biomass obtained. The results indicate that selecting suitable sorbents can support remediation by improving plant growth conditions and, depending on the intended objective, promoting phytostabilization or phytoextraction. Future studies should include a broader range of plant species and examine the most promising sorbents, such as molecular sieve. Furthermore, future research should include trace-element speciation analyses to provide a more comprehensive understanding of metal immobilization mechanisms in soil and to assess the effectiveness of the applied remediation materials more accurately.

## Figures and Tables

**Table 1:** Summary of physicochemical and chemical analyses performed on soil and plant biomass.

Parameter	Material	Method	Instrumentation
Particle-size distribution	Soil	Laser diffraction	Mastersizer 3000, Malvern Instruments, Malvern, UK
Organic carbon	Soil	Elemental analysis	Vario MaxCube CN, Elementar, DE, Langenselbold, Germany
Total nitrogen	Soil	Elemental analysis	Vario MaxCube CN, Elementar, DE
pH (H_2_O, KCl)	Soil	Potentiometric measurement in a 1:2.5 soil: solution suspension (H_2_O and 1 mol KCl dm^−3^) using a glass electrode	HI 2221, Hanna Instruments, Bedfordshire, UK
Hydrolytic acidity (HAC)	Soil	Kappen’s method [86]	Classical titrimetric analysis
Sum of exchangeable base cations (EBCs)	Soil	Kappen’s method [86]	Classical titrimetric analysis
Cd, Pb, Cr, Cu, Zn, Ni, Fe, Mn	Soil, aboveground parts, roots	Inductively coupled plasma optical emission spectrometry (ICP–OES)	iCAP 7000 Series, Thermo Scientific, Waltham, MA, USA

**Table 2:** Equations used to calculate cadmium toxicity, translocation, heavy metal bioaccumulation factors, and heavy metal uptake by *Zea mays.*

Parameter	Equation	Explanations
Cadmium toxicity index (TE)	$\mathrm{TE}=1-\frac{Y_{\mathrm{Cd}}}{Y_{C}}$	Y_C_—SPAD/yield of *Zea mays* grown in uncontaminated (control) soil; Y_Cd_—SPAD/yield of *Zea mays* grown in cadmium-contaminated soil
Heavy metal translocation factor (TF)	TF=MeA MeR	Me_A_—heavy metal content in the aboveground parts of *Zea mays*; Me_R_—heavy metal content in the roots of *Zea mays*
Heavy metal bioaccumulation factor in the aboveground parts of *Zea mays* (BAF_A_)	${\mathrm{BAF}}_{A}=\frac{{\mathrm{Me}}_{A}}{{\mathrm{Me}}_{\mathrm{Sl}}}$	Me_A_—heavy metal content in the aboveground parts of *Zea mays*; Me_Sl_—heavy metal content in soil
Heavy metal bioaccumulation factor in the roots of *Zea mays* (BAF_R_)	BAFR=MeR MeSl	Me_R_—heavy metal content in the roots of *Zea mays*; Me_Sl_—heavy metal content in soil
Heavy metal uptake by *Zea mays* (U)	U_A_ = YA × Me_A_ + Y_R_ × Me_R_	YA—yield of aboveground parts of *Zea mays*; Me_A_—heavy metal content in aboveground parts of *Zea mays*; Me_R_—heavy metal content in roots of *Zea mays*

**Table 3:** Summary of the statistical tests and data analysis methods used.

Analysis Stage	Test/Method	Purpose
Assessment of data distribution	Shapiro–Wilks Test	Verification of the normality of variable distributions
Analysis of variance	Two-way ANOVA	Assessment of the effects of Cd contamination and sorbent type
Post hoc test	Tukey’s HSD test	Identification of homogeneous groups
Effect size	η^2^ (eta squared)	Assessment of the strength of factor effects in ANOVA
Correlations	Pearson’s r	Assessment of relationships between quantitative variables
Data visualization	Boxplots, heatmaps, circular stacked bar chart	Graphical presentation of results and relationships

## Data Availability

The original contributions presented in this study are included in the article, Appendix A, and Supplementary Material. Further inquiries can be directed to the corresponding author.

## Supplementary Materials

The following supporting information can be downloaded at: https://www.mdpi.com/article/10.3390/molecules31203784/s1. Figure S1: SPAD (**a**), yield of the aboveground parts (**b**), and root yield (**c**) of *Zea mays*, g pot^−1^. Figure S2. Schematic comparison of the effects of mineral sorbents on Cd uptake by *Zea mays*. Percentage changes were calculated from mean Cd uptake values across all three soil Cd treatments (0, 15, and 30 mg kg^−1^), relative to the corresponding mean for soil without sorbent addition (Table S1). Table S1: Heavy metal uptake (U) by *Zea mays.*

## Abbreviations

The following abbreviations are used in this manuscript:

SPAD	Leaf greenness index
YA	Yield of the aboveground parts of *Zea mays*
YR	Root yield of *Zea mays*
0	Soil uncontaminated with cadmium
C	Soil not fertilized with mineral sorbents
E	Soil fertilized with expanded clay
Ca	Soil fertilized with calcium carbonate
Z	Soil fertilized with zeolite
S	Soil fertilized with sepiolite
MS	Soil fertilized with molecular sieve
TE	Cadmium toxicity index
BAF_A_	Heavy metal bioaccumulation factor in the aboveground parts of *Zea mays*
BAF_R_	Heavy metal bioaccumulation factor in the roots of *Zea mays*
U	Heavy metal uptake by *Zea mays*
Sl	Soil
A	Aboveground parts
R	Roots
TF	Heavy metal translocation factor

## Appendix A

**Table A1:** Chemical and physicochemical properties of soil.

Treatment	Dose Cd, mg kg^–1^ Soil			Average
	0	15	30	
C_org_, g kg^−1^ soil				
Control (C)	10.568 ± 0.141 ^i C^	10.561 ± 0.050 ^i B^	10.522 ± 0.080 ^i D^	10.550 ^V^
Calcium carbonate (Ca)	10.695 ± 0.037 ^hi C^	10.508 ± 0.092 ^i B^	10.980 ± 0.086 ^fg C^	10.728 ^IV^
Sepiolite (S)	11.618 ± 0.055 ^b A^	11.220 ± 0.073 ^def A^	12.060 ± 0.061 ^a A^	11.633 ^I^
Expanded clay (E)	11.520 ± 0.024 ^bc A^	11.100 ± 0.024 ^efg A^	11.895 ± 0.171 ^a A^	11.505 ^II^
Zeolite (Z)	11.060 ± 0.098 ^fg B^	11.138 ± 0.178 ^def A^	11.385 ± 0.098 ^bcd B^	11.194 ^III^
Molecular sieve (MS)	11.513 ± 0.108 ^bc A^	11.325 ± 0.151 ^cde A^	10.853 ± 0.006 ^gh C^	11.230 ^III^
Average	11.162 ^Y^	10.975 ^X^	11.282 ^Z^	
Source of variation (F statistic and probability level): Cd dose: F—59.53 (*p* < 0.01); Treatment: F—224.73 (*p* < 0.01); Cd dose × Treatment: F—32.50 (*p* < 0.01)				
N_total_, g kg^−1^ soil				
Control (C)	0.852 ± 0.010 ^e AB^	0.852 ± 0.024 ^e BC^	0.831 ± 0.025 ^ef C^	0.845 ^III^
Calcium carbonate (Ca)	0.864 ± 0.013 ^cde A^	0.852 ± 0.016 ^e BC^	0.932 ± 0.016 ^b B^	0.883 ^II^
Sepiolite (S)	0.820 ± 0.003 ^ef B^	0.924 ± 0.036 ^b A^	1.124 ± 0.017 ^a A^	0.956 ^I^
Expanded clay (E)	0.860 ± 0.029 ^cde A^	0.904 ± 0.020 ^bcd AB^	1.096 ± 0.007 ^a A^	0.953 ^I^
Zeolite (Z)	0.744 ± 0.008 ^g C^	0.788 ± 0.003 ^fg D^	0.908 ± 0.010 ^bc B^	0.813 ^IV^
Molecular sieve (MS)	0.856 ± 0.026 ^de AB^	0.840 ± 0.026 ^e CD^	0.916 ± 0.003 ^b B^	0.871 ^II^
Average	0.833 ^X^	0.860 ^Y^	0.968 ^Z^	
Source of variation (F statistic and probability level): Cd dose: F—338.59 (*p* < 0.01); Treatment: F—110.26 (*p* < 0.01); Cd dose × Treatment: F—43.55 (*p* < 0.01)				
pH_KCl_				
Control (C)	4.469 ± 0.020 ^ij E^	4.469 ± 0.020 ^ij B^	4.453 ± 0.010 ^j E^	4.463 ^IV^
Calcium carbonate (Ca)	6.138 ± 0.007 ^d B^	6.257 ± 0.020 ^ab A^	6.288 ± 0.021 ^bc A^	6.227 ^I^
Sepiolite (S)	5.987 ± 0.021 ^e C^	5.901 ± 0.010 ^f B^	5.848 ± 0.036 ^f C^	5.912 ^II^
Expanded clay (E)	4.314 ± 0.032 ^L F^	4.244 ± 0.004 ^m E^	4.524 ± 0.020 ^hi D^	4.361 ^V^
Zeolite (Z)	4.882 ± 0.004 ^g D^	4.377 ± 0.022 ^j D^	4.550 ± 0.008 ^h D^	4.603 ^III^
Molecular sieve (MS)	6.228 ± 0.007 ^bc A^	6.200 ± 0.51 ^bc A^	6.189 ± 0.033 ^cd B^	6.206 ^I^
Average	5.3361 ^Z^	5.241 ^X^	5.308 ^Y^	
Source of variation (F statistic and probability level): Cd dose: F—109.97 (*p* < 0.01); Treatment: F—19,042.31 (*p* < 0.01); Cd dose × Treatment: F—130.18 (*p* < 0.01)				
Hydrolytic acidity, mmol(+) kg^−1^ soil				
Control (C)	31.500 ± 0.306 ^c C^	31.500 ± 0.276 ^c B^	30.000 ± 0.276 ^d C^	31.000 ^III^
Calcium carbonate (Ca)	17.625 ± 0.306 ^ef D^	16.500 ± 0.612 ^gh D^	15.750 ± 0.612 ^h E^	16.625 ^V^
Sepiolite (S)	17.625 ± 0.306 ^ef D^	18.375 ± 0.306 ^e C^	18.375 ± 0.306 ^e D^	18.125 ^IV^
Expanded clay (E)	34.875 ± 0.306 ^a A^	34.125 ± 0.306 ^ab A^	33.375 ± 0.306 ^b A^	34.125 ^I^
Zeolite (Z)	34.125 ± 0.306 ^ab B^	33.375 ± 0.306 ^e A^	31.125 ± 0.306 ^c B^	32.875 ^II^
Molecular sieve (MS)	16.913 ± 0.276 ^fg E^	16.875 ± 0.306 ^fg D^	16.125 ± 0.306 ^gh E^	16.638 ^V^
Average	25.444 ^Z^	25.125 ^Y^	24.125 ^X^	
Source of variation (F statistic and probability level): Cd dose: F—104.84 (*p* < 0.01); Treatment: F—8161.02 (*p* < 0.01); Cd dose × Treatment: F—16.13 (*p* < 0.01)				
Sum of exchangeable base cations, mmol(+) kg^−1^ soil				
Control (C)	122.000 ± 1.633 ^d D^	111.500 ± 2.041 ^hi C^	113.000 ± 2.449 ^gh B^	115.500 ^IV^
Calcium carbonate (Ca)	142.000 ± 1.633 ^a A^	139.000 ± 2.449 ^ab A^	138.000 ± 3.266 ^ab A^	139.667 ^I^
Sepiolite (S)	117.500 ± 0.408 ^defg E^	119.000 ± 0.816 ^def B^	117.000 ± 0.816 ^efg B^	117.833 ^III^
Expanded clay (E)	113.000 ± 2.449 ^gh F^	113.000 ± 0.816 ^gh C^	115.000 ± 0.816 ^fgh B^	113.667 ^IV^
Zeolite (Z)	128.000 ± 1.633 ^c C^	121.500 ± 1.225 ^de B^	108.000 ± 0.816 ^I C^	119.167 ^III^
Molecular sieve (MS)	137.000 ± 2.449 ^b B^	138.000 ± 1.633 ^ab A^	137.500 ± 1.225 ^ab A^	137.500 ^II^
Average	126.583 ^Z^	123.667 ^Y^	121.417 ^X^	
Source of variation (F statistic and probability level): Cd dose: F—51.77 (*p* < 0.01); Treatment: F—515.34 (*p* < 0.01); Cd dose × Treatment: F—26.50 (*p* < 0.01)				
Cation exchange capacity (CEC), mmol(+) kg^−1^ soil				
Control (C)	153.500 ± 1.633 ^c B^	143.000 ± 2.041 ^ef C^	143.000 ± 2.449 ^ef C^	146.500 ^IV^
Calcium carbonate (Ca)	159.625 ± 1.939 ^ab A^	155.500 ± 3.062 ^bc A^	153.750 ± 2.654 ^c A^	156.292 ^I^
Sepiolite (S)	135.125 ± 0.714 ^g D^	137.375 ± 0.510 ^g D^	135.375 ± 0.510 ^g D^	135.958 ^V^
Expanded clay (E)	147.875 ± 2.756 ^d C^	147.125 ± 0.510 ^de B^	148.375 ± 1.123 ^d B^	147.792 ^IV^
Zeolite (Z)	162.125 ± 1.327 ^a A^	154.875 ± 0.919 ^c A^	139.125 ± 0.510 ^fg D^	152.042 ^III^
Molecular sieve (MS)	153.913 ± 2.725 ^c B^	154.875 ± 1.939 ^c A^	153.625 ± 1.531 ^c A^	154.138 ^II–III^
Average	152.027 ^Z^	148.792 ^Y^	145.542 ^X^	
Source of variation (F statistic and probability level): Cd dose: F—76.28 (*p* < 0.01); Treatment: F—193.21 (*p* < 0.01); Cd dose ×Treatment: F—29.83 (*p* < 0.01)				
Base saturation, %				
Control (C)	79.479 ± 0.218 ^d^	77.972 ± 0.315 ^fg C^	79.021 ± 0.360 ^de C^	78.824 ^III^
Calcium carbonate (Ca)	88.958 ± 0.058 ^b A^	89.389 ± 0.185 ^ab A^	89.756 ± 0.575 ^a A^	89.368 ^I^
Sepiolite (S)	86.957 ± 0.158 ^c B C^	86.624 ± 0.273 ^c B^	86.427 ± 0.277 ^c B^	86.669 ^II^
Expanded clay (E)	76.416 ± 0.233 ^h E^	76.805 ± 0.289 ^h D^	77.506 ± 0.036 ^g D^	76.909 ^V^
Zeolite (Z)	78.951 ± 0.361 ^de D^	78.450 ± 0.326 ^ef C^	77.628 ± 0.302 ^g D^	78.343 ^IV^
Molecular sieve (MS)	89.012 ± 0.016 ^b A^	89.104 ± 0.061 ^ab A^	89.504 ± 0.095 ^ab A^	89.206 ^I^
Average	83.295 ^Z^	83.058 ^Y^	83.307 ^Z^	
Source of variation (F statistic and probability level): Cd dose: F—6.50 (*p* < 0.01); Treatment: F—5600.63 (*p* < 0.01); Cd dose × Treatment: F—16.94 (*p* < 0.01)				

Lowercase letters indicate significant differences for both variables, i.e., Cd dose and mineral amendments. Uppercase letters indicate significant differences among sorbent types separately for each Cd dose. Roman numerals indicate significant differences among mean values for mineral amendments, irrespective of Cd dose, and the letters X, Y, and Z indicate significant differences among mean values for Cd doses, irrespective of mineral amendments.

**Table A2:** Heavy metal contents in soils, mg kg^−1^ d.m.

Treatment	Dose Cd, mg kg^–1^ Soil			Average
	0	15	30	
Cd				
Control (C)	0.12 ± 0.01 ^c A^	14.03 ± 0.49 ^b A^	28.05 ± 1.22 ^a A^	14.07 ^I^
Calcium carbonate (Ca)	0.12 ± 0.01 ^c A^	14.46 ± 0.25 ^b A^	28.38 ± 0.65 ^a A^	14.32 ^I^
Sepiolite (S)	0.13 ± 0.03 ^c A^	14.51 ± 0.35 ^b A^	27.77 ± 0.76 ^a A^	14.14 ^I^
Expanded clay (E)	0.11 ± 0.02 ^c A^	14.87 ± 0.61 ^b A^	27.28 ± 1.02 ^a A^	14.09 ^I^
Zeolite (Z)	0.13 ± 0.01 ^c A^	14.40 ± 0.16 ^b A^	28.51 ± 0.74 ^a A^	14.35 ^I^
Molecular sieve (MS)	0.12 ± 0.01 ^c A^	14.16 ± 0.58 ^b A^	28.45 ± 0.72 ^a A^	14.24 ^I^
Average	0.12 ^X^	14.41 ^Y^	28.07 ^Z^	
Source of variation (F statistic and probability level): Cd dose: F—14,685.94 (*p* < 0.01); Treatment: F—0.54(*p* = 0.74); Cd dose × Treatment: F—1.71 (*p* = 0.10)				
Pb				
Control (C)	16.59 ± 0.08 ^ab AB^	16.04 ± 0.03 ^ef B^	16.03 ± 0.02 ^ef D^	16.22 ^III^
Calcium carbonate (Ca)	16.79 ± 0.11 ^a A^	16.57 ± 0.03 ^ab A^	16.55 ± 0.13 ^abc A^	16.64 ^I^
Sepiolite (S)	16.06 ± 0.12 ^ef C^	16.19 ± 0.08 ^ef B^	16.09 ± 0.07 ^ef C^	16.11 ^III^
Expanded clay (E)	16.51 ± 0.09 ^bc B^	16.55 ± 0.08 ^abc A^	16.49 ± 0.08 ^bcd AB^	16.52 ^I–II^
Zeolite (Z)	16.73 ± 0.18 ^ab AB^	16.24 ± 0.16 ^def B^	16.30 ± 0.14 ^cde BC^	16.42 ^II^
Molecular sieve (MS)	16.23 ± 0.02 ^def C^	15.45 ± 0.12 ^g C^	16.64 ± 0.08 ^ab A^	16.11 ^III^
Average	16.49 ^Z^	16.17 ^X^	16.35 ^Y^	
Source of variation (F statistic and probability level): Cd dose: F—55.90 (*p* < 0.01); Treatment: F—56.79 (*p* < 0.01); Cd dose × Treatment: F—32.18 (*p* < 0.01)				
Cr				
Control (C)	44.09 ± 0.05 ^fg B^	44.07 ± 0.02 ^fg E^	44.18 ± 0.02 ^ef C^	44.11 ^IV^
Calcium carbonate (Ca)	43.53 ± 0.04 ^h C^	44.72 ± 0.08 ^ab A^	44.59 ± 0.05 ^bc B^	44.28 ^II^
Sepiolite (S)	43.33 ± 0.08 ^i D^	44.54 ± 0.06 ^c B^	44.77 ± 0.07 ^a A^	44.21 ^III^
Expanded clay (E)	43.54 ± 0.03 ^h C^	44.32 ± 0.08 ^d C^	44.04 ± 0.03 ^c D^	43.97 ^V^
Zeolite (Z)	44.46 ± 0.08 ^c A^	44.26 ± 0.05 ^de CD^	44.51 ± 0.02 ^c B^	44.41 ^I^
Molecular sieve (MS)	44.14 ± 0.03 ^efg B^	44.13 ± 0.03 ^efg DE^	44.06 ± 0.01 ^fg D^	44.11 ^IV^
Average	43.85 ^Z^	44.34 ^Y^	44.36 ^Y^	
Source of variation (F statistic and probability level): Cd dose: F—729.30 (*p* < 0.01); Treatment: F—104.11 (*p* < 0.01); Cd dose × Treatment: F—203.95 (*p* < 0.01)				
Cu				
Control (C)	13.15 ± 0.04 ^def AB^	13.15 ± 0.04 ^def D^	12.74 ± 0.11 ^hi C^	13.01 ^III^
Calcium carbonate (Ca)	12.81 ± 0.08 ^ghi B^	13.74 ± 0.03 ^ab A^	12.65 ± 0.03 ^i C^	13.07 ^III^
Sepiolite (S)	13.02 ± 0.05 ^fgh B^	13.04 ± 0.05 ^efh D^	12.87 ± 0.04 ^fghi BC^	12.98 ^III^
Expanded clay (E)	13.02 ± 0.07 ^fgh B^	13.51 ± 0.06 ^bc B^	13.54 ± 0.23 ^abc A^	13.36 ^II^
Zeolite (Z)	13.52 ± 0.12 ^bc A^	13.35 ± 0.08 ^cde C^	13.12 ± 011 ^defg B^	13.33 ^II^
Molecular sieve (MS)	13.43 ± 0.39 ^bcd A^	13.85 ± 0.04 ^a A^	13.61 ± 0.08 ^abc A^	13.63 ^I^
Average	13.16 ^Y^	13.44 ^Z^	13.09 ^Y^	
Source of variation (F statistic and probability level): Cd dose: F—52.44 (*p* < 0.01); Treatment: F—49.11 (*p* < 0.01); Cd dose ×Treatment: F—18.58 (*p* < 0.01)				
Zn				
Control (C)	53.56 ± 0.07 ^f D^	54.06 ± 0.06 ^de C^	54.18 ± 0.08 ^de B^	53.93 ^III^
Calcium carbonate (Ca)	55.05 ± 0.06 ^a A^	54.41 ± 0.05 ^cd BC^	54.38 ± 0.08 ^cd B^	54.61 ^I^
Sepiolite (S)	54.93 ± 0.08 ^ab A^	54.40 ± 0.38 ^cd BC^	54.91 ± 0.17 ^ab A^	54.75 ^I^
Expanded clay (E)	54.57 ± 0.01 ^bc B^	54.62 ± 0.03 ^bc AB^	55.07 ± 0.06 ^a A^	54.75 ^I^
Zeolite (Z)	54.05 ± 0.10 ^de C^	54.05 ± 0.12 ^de C^	54.12 ± 0.08 ^de B^	54.07 ^III^
Molecular sieve (MS)	53.95 ± 0.01 ^e C^	54.85 ± 0.12 ^ab A^	54.19 ± 0.36 ^de B^	54.32 ^II^
Average	54.35 ^Y^	54.40 ^ZY^	54.47 ^Z^	
Source of variation (F statistic and probability level): Cd dose: F—4.18 (*p* =0.02); Treatment: F—71.79 (*p* < 0.01); Cd dose × Treatment: F—23.71 (*p* < 0.01)				
Ni				
Control (C)	10.19 ± 0.01 ^f C^	10.20 ± 0.04 ^ef BC^	10.17 ± 0.02 ^f D^	10.19 ^IV^
Calcium carbonate (Ca)	10.18 ± 0.02 ^f C^	10.12 ± 0.01 ^f C^	10.45 ± 0.04 ^bc AB^	10.25 ^III^
Sepiolite (S)	10.16 ± 0.03 ^f C^	10.23 ± 0.06 ^ef B^	10.19 ± 0.08 ^f CD^	10.19 ^IV^
Expanded clay (E)	10.45 ± 0.04 ^bc B^	10.18 ± 0.04 ^f BC^	10.32 ± 0.07 ^de BC^	10.32 ^II^
Zeolite (Z)	10.71 ± 0.08 ^a A^	10.45 ± 0.05 ^bc A^	10.53 ± 0.08 ^b A^	10.56 ^I^
Molecular sieve (MS)	10.19 ± 0.02 ^f C^	10.41 ± 0.02 ^cd A^	10.45 ± 0.02 ^bc AB^	10.35 ^II^
Average	10.31 ^Y^	10.27 ^X^	10.35 ^Z^	
Source of variation (F statistic and probability level): Cd dose: F—20.54 (*p* < 0.01); Treatment: F—107.37 (*p* < 0.01); Cd dose × Treatment: F—28.09 (*p* < 0.01)				
Fe				
Control (C)	12,704.62 ± 45.13 ^cd BC^	12,788.18 ± 81.65 ^bc A^	12,737.09 ± 81.65 ^cd B^	12,743.30 ^I^
Calcium carbonate (Ca)	12,781.71 ± 76.55 ^bc B^	12,485.19 ± 47.23 ^ef B^	12,591.85 ± 44.45 ^de B^	12,619.58 ^II^
Sepiolite (S)	12,215.23 ± 16.57 ^g D^	12,362.14 ± 51.01 ^fg BC^	12,644.98 ± 82.83 ^cde B^	12,407.45 ^III^
Expanded clay (E)	12,243.22 ± 60.00 ^g D^	12,717.63 ± 81.95 ^cd A^	12,705.61 ± 41.74 ^cd B^	12,555.49 ^II^
Zeolite (Z)	12,607.31 ± 67.86 ^de C^	12,726.55 ± 35.90 ^cd A^	12,967.04 ± 31.14 ^a A^	12,766.97 ^I^
Molecular sieve (MS)	12,943.05 ± 62.86 ^g A^	12,270.76 ± 57.41 ^g C^	12,628.38 ± 98.43 ^cde B^	12,614.06 ^II^
Average	12,582.52 ^Y^	12,558.41 ^Y^	12,712.49 ^Z^	
Source of variation (F statistic and probability level): Cd dose: F—41.78 (*p* < 0.01); Treatment: F—52.39 (*p* < 0.01); Cd dose ×Treatment: F—50.83 (*p* < 0.01)				
Mn				
Control (C)	230.19 ± 0.69 ^cde C^	225.93 ± 0.82 ^efg C^	220.64 ± 3.23 ^gh B^	225.59 ^III^
Calcium carbonate (Ca)	229.90 ± 1.43 ^def C^	223.28 ± 2.19 ^fgh C^	219.14 ± 0.82 ^h B^	224.19 ^III^
Sepiolite (S)	236.93 ± 1.63 ^abcd AB^	237.91 ± 0.54 ^ab B^	236.25 ± 4.18 ^abcd A^	237.03 ^I–II^
Expanded clay (E)	239.52 ± 0.36 ^ab A^	240.94 ± 1.84 ^ab A^	236.22 ± 1.73 ^abc A^	238.89 ^I–II^
Zeolite (Z)	236.25 ± 0.52 ^abcd B^	240.57 ± 0.44 ^ab AB^	240.72 ± 4.08 ^ab A^	239.18 ^I^
Molecular sieve (MS)	234.85 ± 2.06 ^abcd B^	238.66 ± 0.36 ^ab BC^	234.10 ± 7.48 ^bcd A^	235.87 ^II^
Average	234.61 ^Z^	234.55 ^Z^	231.18 ^Y^	
Source of variation (F statistic and probability level): Cd dose: F—13.49 (*p* < 0.01); Treatment: F—8064 (*p* < 0.01); Cd dose × Treatment: F—5.63 (*p* < 0.01)				

Lowercase letters indicate significant differences for both variables, i.e., Cd dose and mineral amendments. Uppercase letters indicate significant differences among sorbent types separately for each Cd dose. Roman numerals indicate significant differences among mean values for mineral amendments, irrespective of Cd dose, and the letters X, Y, and Z indicate significant differences among mean values for Cd doses, irrespective of mineral amendments.

**Table A3:** Heavy metal contents in the aboveground parts of *Zea mays*, mg kg^−1^ d.m.

Treatment	Dose Cd, mg kg^–1^ Soil			Average
	0	15	30	
Cd				
Control (C)	0.015 ± 0.001 ^j A^	0.300 ± 0.044 ^h D^	0.923 ± 0.002 ^c C^	0.413 ^III^
Calcium carbonate (Ca)	0.003 ± 0.001 ^j D^	0.305 ± 0.001 ^h D^	0.773 ± 0.001 ^d D^	0.360 ^V^
Sepiolite (S)	0.010 ± 0.001 ^j BC^	0.378 ± 0.001 ^g C^	0.738 ± 0.001 ^e E^	0.375 ^IV^
Expanded clay (E)	0.009 ± 0.001 ^j C^	0.572 ± 0.001 ^f B^	1.642 ± 0.003 ^b B^	0.741 ^II^
Zeolite (Z)	0.010 ± 0.001 ^j BC^	0.718 ± 0.001 ^e A^	1.830 ± 0.001 ^a A^	0.853 ^I^
Molecular sieve (MS)	0.011 ± 0.001 ^j B^	0.094 ± 0.001 ^I E^	0.120 ± 0.001 ^i F^	0.075 ^VI^
Average	0.010 ^X^	0.394 ^Y^	1.004 ^Z^	
Source of variation (F statistic and probability level): Cd dose: F—54,746.71 (*p* < 0.01); Treatment: F—8714.34 (*p* < 0.01); Cd dose × Treatment: F—3789.76 (*p* < 0.01)				
Pb				
Control (C)	0.750 ± 0.100 ^efgh B^	1.400 ± 0.163 ^a A^	0.550 ± 0.100 ^hi D^	0.900 ^I–II^
Calcium carbonate (Ca)	1.150 ± 0.100 ^b A^	0.950 ± 0.058 ^bcde CD^	0.800 ± 0.082 ^defg BC^	0.967 ^I^
Sepiolite (S)	0.750 ± 0.058 ^efgh B^	0.800 ± 0.041 ^defg D^	1.025 ± 0.050 ^bc A^	0.858 ^II–III^
Expanded clay (E)	0.600 ± 0.041 ^ghi B^	1.150 ± 0.058 ^b B^	0.550 ± 0.058 ^hi D^	0.767 ^III^
Zeolite (Z)	1.150 ± 0.058 ^b A^	0.500 ± 0.082 ^I E^	0.650 ± 0.100 ^fghi CD^	0.767 ^III^
Molecular sieve (MS)	1.000 ± 0.082 ^bcd A^	1.000 ± 0.005 ^bcd BC^	0.850 ± 0.058 ^cdef B^	0.950 ^I–II^
Average	0.900 ^Y^	0.967 ^Z^	0.738 ^X^	
Source of variation (F statistic and probability level): Cd dose: F—53.36 (*p* < 0.01); Treatment: F—14.64 (*p* < 0.01); Cd dose × Treatment: F—51.28 (*p* < 0.01)				
Cr				
Control (C)	4.200 ± 0.566 ^a A^	3.400 ± 0.163 ^b A^	2.800 ± 0.327 ^cde A^	3.467 ^I^
Calcium carbonate (Ca)	2.600 ± 0.141 ^de C^	2.850 ± 0.100 ^cde BC^	1.950 ± 0.100 ^fg B^	2.467 ^IV^
Sepiolite (S)	2.650 ± 0.238 ^de C^	2.800 ± 0.082 ^cde C^	2.500 ± 0.115 ^e A^	2.650 ^IV^
Expanded clay (E)	3.200 ± 0.283 ^bc BC^	1.800 ± 0.141 ^g E^	1.600 ± 0.163 ^g B^	2.200 ^V^
Zeolite (Z)	3.550 ± 0.058 ^b B^	2.400 ± 0.163 ^ef D^	2.550 ± 0.100 ^e A^	2.833 ^III^
Molecular sieve (MS)	3.600 ± 0.005 ^b AB^	3.100 ± 0.115 ^bcd B^	2.750 ± 0.208 ^cde A^	3.150 ^II^
Average	3.300 ^Z^	2.725 ^Y^	2.358 ^X^	
Source of variation (F statistic and probability level): Cd dose: F—122.68 (*p* < 0.01); Treatment: F—57.80 (*p* < 0.01); Cd dose × Treatment: F—12.95 (*p* < 0.01)				
Cu				
Control (C)	2.950 ± 0.100 ^fgh B^	3.400 ± 0.283 ^de B^	4.050 ± 0.100 ^c B^	3.467 ^II^
Calcium carbonate (Ca)	2.400 ± 0.283 ^ij C^	3.050 ± 0.191 ^efg BC^	2.800 ± 0.283 ^fghi D^	2.750 ^IV^
Sepiolite (S)	2.000 ± 0.010 ^j D^	2.700 ± 0.115 ^ghi CD^	3.600 ± 0.010 ^d C^	2.767 ^IV^
Expanded clay (E)	3.050 ± 0.100 ^efg B^	3.150 ± 0.191 ^ef B^	3.750 ± 0.100 ^cd BC^	3.317 ^II^
Zeolite (Z)	3.450 ± 0.191 ^de A^	2.600 ± 0.010 ^hi D^	2.900 ± 0.115 ^fgh D^	2.983 ^III^
Molecular sieve (MS)	2.600 ± 0.010 ^hi C^	6.150 ± 0.100 ^a A^	4.950 ± 0.010 ^b A^	4.567 ^I^
Average	2.742 ^X^	3.508 ^Y^	3.675 ^Z^	
Source of variation (F statistic and probability level): Cd dose: F—231.02 (*p* < 0.01); Treatment: F—216.25 (*p* < 0.01); Cd dose × Treatment: F—65.21 (*p* < 0.01)				
Zn				
Control (C)	17.400 ± 0.082 ^k A^	34.850 ± 0.100 ^c B^	32.600 ± 0.100 ^d B^	28.283 ^II^
Calcium carbonate (Ca)	12.850 ± 0.100 ^p E^	19.750 ± 0.100 ^g D^	18.750 ± 0.100 ^I D^	17.117 ^V^
Sepiolite (S)	15.000 ± 0.010 ^n D^	13.700 ± 0.115 ^o F^	11.800 ± 0.010 ^r F^	13.500 ^VI^
Expanded clay (E)	15.400 ± 0.100 ^m C^	44.800 ± 0.100 ^a A^	41.600 ± 0.010 ^b A^	33.933 ^I^
Zeolite (Z)	15.400 ± 0.010 ^m C^	28.600 ± 0.100 ^e C^	26.600 ± 0.010 ^f C^	23.533 ^III^
Molecular sieve (MS)	17.000 ± 0.10 ^L B^	19.200 ± 0.100 ^h E^	18.600 ± 0.010 ^j E^	18.267 ^IV^
Average	15.508 ^X^	26.817 ^Z^	24.992 ^Y^	
Source of variation (F statistic and probability level): Cd dose: F—298,542.56 (*p* < 0.01); Treatment: F—237,423.00 (*p* < 0.01); Cd dose × Treatment: F—53,333.51 (*p* < 0.01)				
Ni				
Control (C)	2.700 ± 0.115 ^b A^	4.000 ± 0.041 ^a A^	2.350 ± 0.100 ^c A^	3.017 ^I^
Calcium carbonate (Ca)	1.650 ± 0.100 ^fg C^	1.800 ± 0.010 ^efg BC^	2.000 ± 0.010 ^de B^	1.817 ^III^
Sepiolite (S)	1.650 ± 0.191 ^fg C^	1.850 ± 0.100 ^efg B^	2.050 ± 0.191 ^cde B^	1.850 ^III^
Expanded clay (E)	1.850 ± 0.191 ^efg BC^	1.550 ± 0.191 ^gC^	2.000 ± 0.010 ^de B^	1.800 III
Zeolite (Z)	2.000 ± 0.010 ^de B^	1.850 ± 0.100 ^efg B^	1.900 ± 0.115 ^ef B^	1.917 ^II–III^
Molecular sieve (MS)	1.800 ± 0.010 ^efg BC^	2.050 ± 0.191 ^cde B^	2.300 ± 0.115 ^cd A^	2.050 ^II^
Average	1.942 ^Y^	2.183 ^Z^	2.100 ^Z^	
Source of variation (F statistic and probability level): Cd dose: F—24.72 (*p* < 0.01); Treatment: F—181.34 (*p* < 0.01); Cd dose ×Treatment: F—46.87 (*p* < 0.01)				
Fe				
Control (C)	132.850 ± 0.100 ^fg B^	148.550 ± 3.206 ^de D^	220.550 ± 2.734 ^a A^	167.317 ^I^
Calcium carbonate (Ca)	128.350 ± 1.792 ^fgh C^	160.500 ± 2.169 ^c C^	175.200 ± 4.899 ^b B^	154.683 ^II^
Sepiolite (S)	120.600 ± 1.633 ^h D^	173.850 ± 2.074 ^b B^	221.400 ± 16.330 ^a A^	171.950 ^I^
Expanded clay (E)	137.400 ± 3.266 ^ef A^	159.800 ± 3.266 ^cd C^	147.000 ± 0.473 ^e C^	148.067 ^III^
Zeolite (Z)	121.600 ± 1.633 ^gh D^	180.850 ± 2.452 ^b A^	147.000 ± 3.394 ^e C^	149.817 ^II–III^
Molecular sieve (MS)	134.950 ± 0.173 ^f AB^	118.800 ± 1.633 ^h E^	119.750 ± 1.038 ^h D^	124.500 ^IV^
Average	129.292 ^X^	157.058 ^Y^	171.817 ^Z^	
Source of variation (F statistic and probability level): Cd dose: F—546.07 (*p* < 0.01); Treatment: F—165.53 (*p* < 0.01); Cd dose × Treatment: F—140.51 (*p* < 0.01)				
Mn				
Control (C)	70.550 ± 0.661 ^j C^	145.000 ± 3.266 ^c B^	136.250 ± 1.509 ^d C^	117.267 ^II^
Calcium carbonate (Ca)	75.350 ± 0.100 ^h A^	84.250 ± 0.100 ^f D^	83.050 ± 0.199 ^fg D^	80.883 ^IV^
Sepiolite (S)	71.800 ± 0.566 ^j B^	74.750 ± 0.342 ^hi E^	81.350 ± 1.886 ^g D^	75.967 ^V^
Expanded clay (E)	71.750 ± 0.473 ^j BC^	159.100 ± 0.945 ^a A^	153.600 ± 0.283 ^b A^	128.150 ^I^
Zeolite (Z)	72.300 ± 0.663 ^ij B^	122.650 ± 0.100 ^e C^	147.55 ± 0.943 ^c B^	114.167 ^III^
Molecular sieve (MS)	30.400 ± 0.566 ^k D^	25.450 ± 0.100 ^L F^	23.900 ± 0.200 ^L E^	26.583 ^VI^
Average	65.358 ^X^	101.867 ^Y^	104.283 ^Z^	
Source of variation (F statistic and probability level): Cd dose: F—10,103.79 (*p* < 0.01); Treatment: F—15,046.27 (*p* < 0.01); Cd dose × Treatment: F—1928.66 (*p* < 0.01)				

Lowercase letters indicate significant differences for both variables, i.e., Cd dose and mineral amendments. Uppercase letters indicate significant differences among sorbent types separately for each Cd dose. Roman numerals indicate significant differences among mean values for mineral amendments, irrespective of Cd dose, and the letters X, Y, and Z indicate significant differences among mean values for Cd doses, irrespective of mineral amendments.

**Table A4:** Heavy metal contents in the roots of *Zea mays*, mg kg^−1^ d.m.

Treatment	Dose Cd, mg kg^–1^ Soil			Average
	0	15	30	
Cd				
Control (C)	0.050 ± 0.004 ^L A^	5.512 ± 0.096 ^b A^	6.151 ± 0.011 ^a A^	3.904 ^I^
Calcium carbonate (Ca)	0.034 ± 0.001 ^L C^	2.012 ± 0.004 ^I E^	3.170 ± 0.002 ^g E^	1.739 ^IV^
Sepiolite (S)	0.051 ± 0.001 ^L A^	2.893 ± 0.005 ^h D^	3.244 ± 0.005 ^f D^	2.063 ^III^
Expanded clay (E)	0.043 ± 0.001 ^L AB^	3.535 ± 0.008 ^e B^	4.776 ± 0.005 ^d C^	2.784 ^II^
Zeolite (Z)	0.034 ± 0.001 ^L C^	3.170 ± 0.007 ^g C^	5.149 ± 0.012 ^c B^	2.784 ^II^
Molecular sieve (MS)	0.037 ± 0.008 ^L BC^	0.743 ± 0.016 ^k F^	1.011 ± 0.003 ^j F^	0.597 ^V^
Average	0.041 ^X^	2.978 ^Y^	3.917 ^Z^	
Source of variation (F statistic and probability level): Cd dose: F—175,416.73 (*p* < 0.01); Treatment: F—27,100.23 (*p* < 0.01); Cd dose × Treatment: F—7484.28 (*p* < 0.01)				
Pb				
Control (C)	2.000 ± 0.082 ^a A^	1.700 ± 0.082 ^b A^	1.400 ± 0.016 ^cd A^	1.700 ^I^
Calcium carbonate (Ca)	1.350 ± 0.058 ^d C^	0.700 ± 0.082 ^ij D^	1.000 ± 0.082 ^fg B^	1.017 ^IV^
Sepiolite (S)	1.050 ± 0.058 ^ef D^	1.250 ± 0.058 ^de B^	1.100 ± 0.115 ^ef B^	1.133 ^III^
Expanded clay (E)	1.050 ± 0.058 ^ef D^	0.900 ± 0.082 ^fghi C^	0.600 ± 0.082 ^j D^	0.850 ^V^
Zeolite (Z)	1.600 ± 0.082 ^bc B^	0.950 ± 0.058 ^fgh C^	0.800 ± 0.082 ^ghij C^	1.117 ^III^
Molecular sieve (MS)	1.600 ± 0.082 ^bc B^	1.600 ± 0.010 ^bc A^	0.750 ± 0.058 ^hij CD^	1.317 ^II^
Average	1.442 ^Z^	1.183 ^Y^	0.942 ^X^	
Source of variation (F statistic and probability level): Cd dose: F—225.08 (*p* < 0.01); Treatment: F—154.93 (*p* < 0.01); Cd dose × Treatment: F—36.68 (*p* < 0.01)				
Cr				
Control (C)	14.800 ± 0.712 ^a A^	8.800 ± 0.653 ^c A^	4.200 ± 0.245 ^ghi B^	9.267 ^I^
Calcium carbonate (Ca)	8.100 ± 0.416 ^cd C^	4.950 ± 0.823 ^fghi B^	3.150 ± 0.526 ^i B^	5.400 ^II–III^
Sepiolite (S)	7.150 ± 0.806 ^cde CD^	4.250 ± 0.191 ^ghi B^	3.650 ± 0.379 ^hi B^	5.017 ^III^
Expanded clay (E)	5.750 ± 0.500 ^efg D^	5.100 ± 0.577 ^fgh B^	4.300 ± 0.622 ^ghi B^	5.050 ^III^
Zeolite (Z)	8.550 ± 0.342 ^cd C^	5.050 ± 0.661 ^fgh B^	4.200 ± 0.490 ^ghi B^	5.933 ^II^
Molecular sieve (MS)	11.600 ± 1.633 ^b B^	8.800 ± 0.327 ^c A^	6.750 ± 1.088 ^def A^	9.050 ^I^
Average	9.325 ^Z^	6.158 ^Y^	4.375 ^X^	
Source of variation (F statistic and probability level): Cd dose: F—306.80 (*p* < 0.01); Treatment: F—97.16 (*p* < 0.01); Cd dose × Treatment: F—19.68 (*p* < 0.01)				
Cu				
Control (C)	3.700 ± 0.200 ^gh B^	4.650 ± 0.342 ^de B^	5.200 ± 0.163 ^bc B^	4.517 ^II^
Calcium carbonate (Ca)	3.400 ± 0.163 ^hi BC^	3.450 ± 0.058 ^hi E^	3.650 ± 0.191 ^gh D^	3.500 ^IV^
Sepiolite (S)	3.550 ± 0.412 ^h B^	4.350 ± 0.058 ^ef BC^	4.350 ± 0.100 ^ef C^	4.083 ^III^
Expanded clay (E)	3.550 ± 0.100 ^h B^	4.050 ± 0.100 ^fgCD^	4.100 ± 0.115 ^fg C^	3.900 ^III^
Zeolite (Z)	3.050 ± 0.100 ^i C^	3.700 ± 0.115 ^gh DE^	5.200 ± 0.163 ^bc B^	3.983 ^III^
Molecular sieve (MS)	4.850 ± 0.100 ^cd A^	5.400 ± 0.327 ^ab A^	5.800 ± 0.163 ^a A^	5.350 ^I^
Average	3.683 ^X^	4.267 ^Y^	4.717 ^Z^	
Source of variation (F statistic and probability level): Cd dose: F—175.70 (*p* < 0.01); Treatment: F—134.86 (*p* < 0.01); Cd dose × Treatment: F—15.97 (*p* < 0.01)				
Zn				
Control (C)	51.400 ± 0.245 ^a A^	28.400 ± 0.163 ^f A^	42.850 ± 0.100 ^d A^	40.883 ^I^
Calcium carbonate (Ca)	43.750 ± 0.191 ^c C^	17.750 ± 0.100 ^m E^	17.450 ± 0.100 ^m E^	26.317 ^III^
Sepiolite (S)	22.150 ± 0.100 ^j F^	13.750 ± 0.100 ^n F^	12.750 ± 0.100 ^o F^	16.217 ^V^
Expanded clay (E)	46.200 ± 0.100 ^b B^	26.800 ± 0.100 ^g B^	19.400 ± 0.163 ^L D^	30.800 ^II^
Zeolite (Z)	31.050 ± 0.100 ^e E^	20.850 ± 0.100 ^k D^	20.750 ± 0.100 ^k C^	24.217 ^IV^
Molecular sieve (MS)	42.600 ± 0.327 ^d D^	25.200 ± 0.163 ^h C^	24.650 ± 0.191 ^I B^	30.817 ^II^
Average	39.525 ^Z^	22.125 ^X^	22.975 ^Y^	
Source of variation (F statistic and probability level): Cd dose: F—128,572.33 (*p* < 0.01); Treatment: F—45,091.13 (*p* < 0.01); Cd dose × Treatment: F—6376.53 (*p* < 0.01)				
Ni				
Control (C)	9.950 ± 0.191 ^a A^	9.200 ± 0.490 ^b A^	8.600 ± 0.490 ^bc A^	9.250 ^I^
Calcium carbonate (Ca)	4.850 ± 0.100 ^fg D^	4.900 ± 0.115 ^fg C^	4.150 ± 0.100 ^h E^	4.633 ^V^
Sepiolite (S)	4.450 ± 0.252 ^gh DE^	5.350 ± 0.100 ^ef C^	5.400 ± 0.163 ^ef D^	5.067 ^IV^
Expanded clay (E)	5.350 ± 0.252 ^ef C^	7.800 ± 0.163 ^d B^	7.500 ± 0.200 ^d C^	6.883 ^III^
Zeolite (Z)	5.950 ± 0.191 ^e B^	7.850 ± 0.191 ^d B^	8.050 ± 0.100 ^cd B^	7.283 ^II^
Molecular sieve (MS)	4.100 ± 0.200 ^h E^	4.850 ± 0.252 ^fg C^	4.350 ± 0.100 ^gh E^	4.433 ^V^
Average	5.775 ^X^	6.658 ^Z^	6.342 ^Y^	
Source of variation (F statistic and probability level): Cd dose: F—88.59 (*p* < 0.01); Treatment: F—786.56 (*p* < 0.01); Cd dose × Treatment: F—44.12 (*p* < 0.01)				
Fe				
Control (C)	2472.550 ± 43.370 ^b A^	1754.400 ± 48.990 ^c A^	1265.200 ± 48.990 ^g C^	1830.717 ^II^
Calcium carbonate (Ca)	2547.400 ± 52.380 ^a A^	1016.000 ± 94.688 ^h C^	632.400 ± 62.847 ^i E^	1398.600 ^V^
Sepiolite (S)	2554.850 ± 38.256 ^a A^	1073.050 ± 10.641 ^h C^	1077.200 ± 11.653 ^h D^	1568.367 ^IV^
Expanded clay (E)	2469.350 ± 37.814 ^b A^	1279.700 ± 21.631 ^g B^	1356.350 ± 5.691 ^f B^	1701.800 ^III^
Zeolite (Z)	2559.750 ± 32.345 ^a A^	1655.850 ± 27.561 ^de A^	1638.150 ± 10.655 ^de A^	1951.250 ^II^
Molecular sieve (MS)	2531.550 ± 64.180 ^ab A^	1691.250 ± 44.340 ^d A^	1606.100 ± 18.861 ^e A^	1942.967 ^II^
Average	2522.575 ^Z^	1411.708 ^Y^	1262.567 ^X^	
Source of variation (F statistic and probability level): Cd dose: F—5962.19 (*p* < 0.01); Treatment: F—303.36 (*p* < 0.01); Cd dose ×Treatment: F—110.74 (*p* < 0.01)				
Mn				
Control (C)	153.400 ± 2.304 ^a A^	145.800 ± 2.286 ^a A^	120.950 ± 1.935 ^bc A^	140.050 ^I^
Calcium carbonate (Ca)	64.800 ± 2.315 ^f D^	93.650 ± 9.241 ^e C^	106.350 ± 11.274 ^d B^	88.267 ^IV^
Sepiolite (S)	67.050 ± 1.248 ^f D^	126.450 ± 1.320 ^b B^	126.250 ± 3.017 ^bc A^	106.583 ^III^
Expanded clay (E)	92.000 ± 1.818 ^e B^	145.400 ± 3.767 ^a A^	119.150 ± 1.159 ^bc A^	118.850 ^II^
Zeolite (Z)	84.000 ± 1.424 ^e C^	127.550 ± 2.511 ^b B^	116.050 ± 1.644 ^cd AB^	109.200 ^III^
Molecular sieve (MS)	43.050 ± 1.893 ^g E^	59.200 ± 1.840 ^f D^	35.900 ± 0.959 ^g C^	46.050 ^V^
Average	84.050 ^X^	116.342 ^Z^	104.108 ^Y^	
Source of variation (F statistic and probability level): Cd dose: F—406.74 (*p* < 0.01); Treatment: F—783.83 (*p* < 0.01); Cd dose × Treatment: F—85.88 (*p* < 0.01)				

Lowercase letters indicate significant differences for both variables, i.e., Cd dose and mineral amendments. Uppercase letters indicate significant differences among sorbent types separately for each Cd dose. Roman numerals indicate significant differences among mean values for mineral amendments, irrespective of Cd dose, and the letters X, Y, and Z indicate significant differences among mean values for Cd doses, irrespective of mineral amendments.
