# Supplementary material for: Interactions Between Cadmium Contamination and Mineral Sorbents in Shaping Heavy Metal Bioaccumulation in Soil and Plant Biomass
Source: Molecules. 2026 Oct 21;31(20):3784. doi: 10.3390/molecules31203784 (PMC13707395; doi:10.3390/molecules31203784)
Supplement: Supplementary file 1 [file molecules-31-03784-s001.zip › molecules-4610956-supplementary.pdf]

molecules

Supplementary Materials

Interactions Between Cadmium Contamination and Mineral Sorbents in Shaping Heavy Metal Bioaccumulation in Soil and Plant Biomass

Jadwiga Wyszkowska *, Agata Borowik, Magdalena Zaborowska, and Jan Kucharski

Department of Soil Science and Microbiology, Faculty of Agriculture and Forestry, University of Warmia and Mazury in Olsztyn, Plac Łódzki 3, 10-727 Olsztyn, Poland; agata.borowik@uwm.edu.pl (A.B.); m.zaborowska@uwm.edu.pl (M.Z.); jan.kucharski@uwm.edu.pl (J.K.)
* Correspondence: jadwiga.wyszkowska@uwm.edu.pl

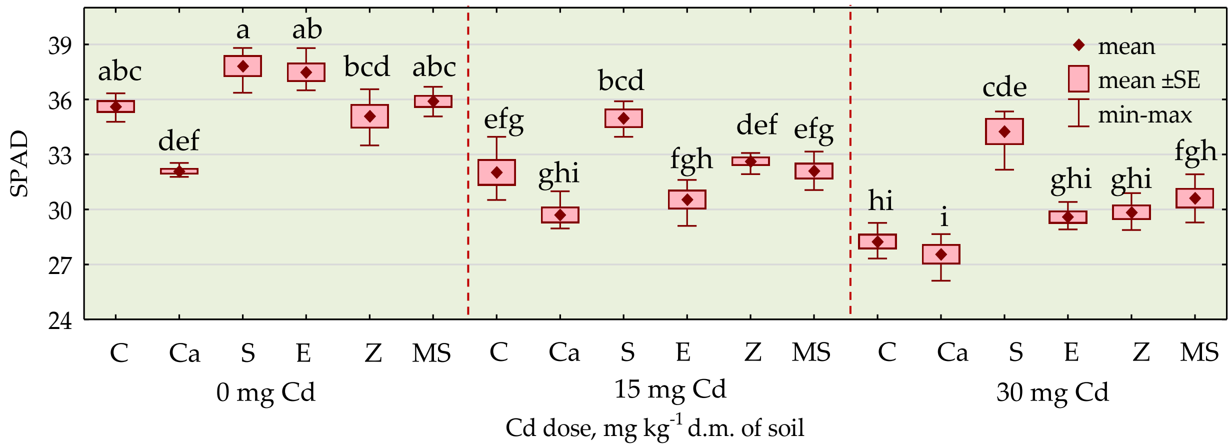

(a)

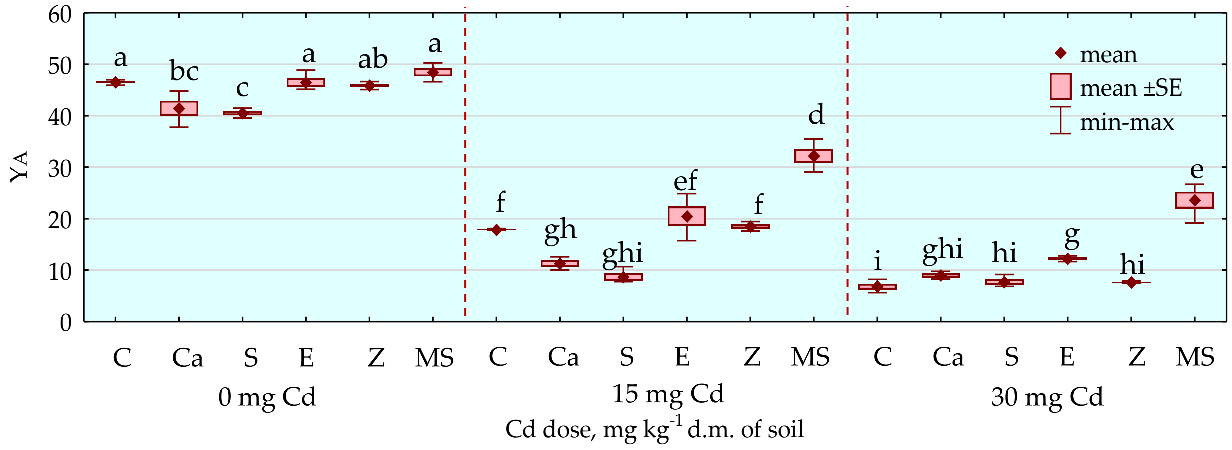

(b)

 https://doi.org/10.3390/xxxxx

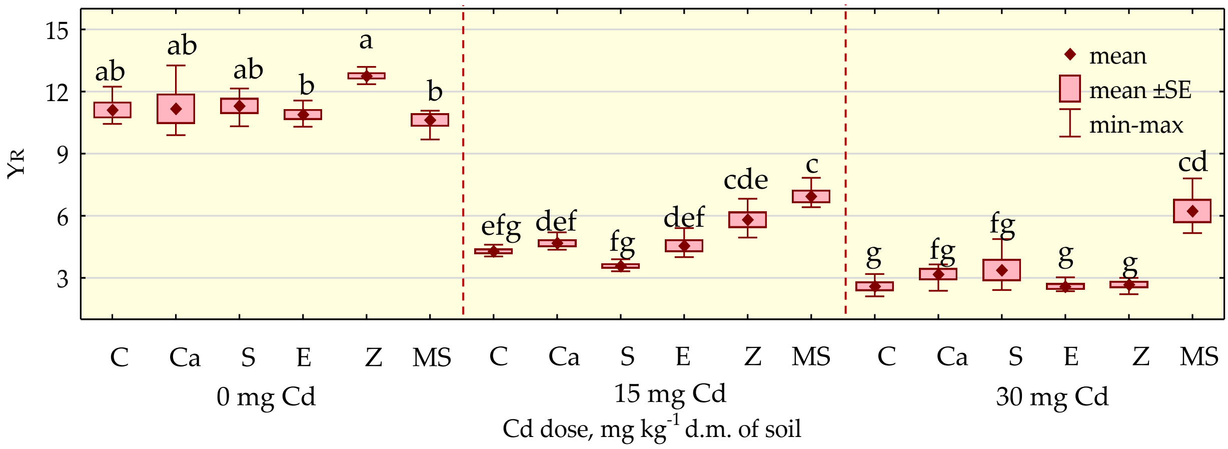

(c)

Figure S1. SPAD (a), yield of the aboveground parts (b), and root yield (c) of *Zea mays*, g pot^{-1}
Lowercase letters indicate significant differences for both variables, i.e., Cd dose and mineral amendments.

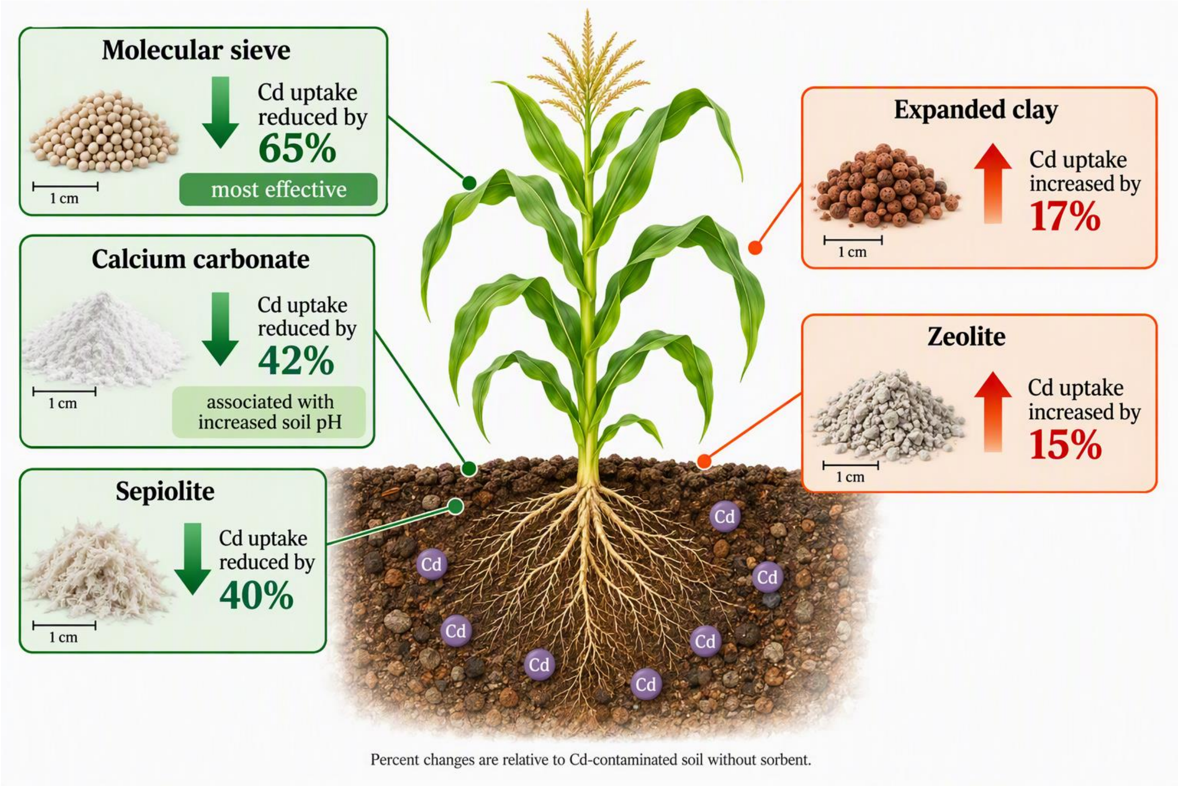

Figure S2. Schematic comparison of the effects of mineral sorbents on Cd uptake by *Zea mays*. Percentage changes were calculated from mean Cd uptake values across all three soil Cd treatments (0, 15, and 30 mg kg^{-1}), relative to the corresponding mean for soil without sorbent addition (Table S1).

Table S1. Heavy metal uptake (U) by *Zea mays*

Treatment	Cadmium dose, mg kg^{-1} d.m. of soil			Average
	0	15	30	
Cd				
Control (C)	1.253 ± 0.060 $^{g\,A}$	28.975 ± 2.163 $^{ab\,AB}$	22.239 ± 2.368 $^{c\,B}$	17.489 ^{II}
Calcium carbonate (Ca)	0.504 ± 0.044 $^{g\,C}$	12.866 ± 0.682 $^{de\,C}$	17.046 ± 1.675 $^{d\,BC}$	10.139 ^{III}
Sepiolite (S)	0.982 ± 0.043 $^{g\,B}$	13.640 ± 0.805 $^{d\,C}$	16.650 ± 1.154 $^{d\,C}$	10.424 ^{III}
Expanded clay (E)	0.887 ± 0.029 $^{g\,B}$	27.816 ± 1.403 $^{b\,B}$	32.456 ± 2.130 $^{a\,A}$	20.386 ^{I}
Zeolite (Z)	0.892 ± 0.015 $^{g\,B}$	31.712 ± 2.483 $^{ab\,A}$	27.829 ± 1.801 $^{b\,A}$	20.145 ^{I}
Molecular sieve (MS)	0.926 ± 0.066 $^{g\,B}$	8.179 ± 0.145 $^{f\,D}$	9.136 ± 1.335 $^{ef\,D}$	6.080 ^{IV}
Average	0.907 ^{Y}	20.531 ^{Z}	20.893 ^{Z}	
Source of variation (F statistic and probability level):				
Cd dose: F – 1148.53 (p <0.01); Treatment: F – 159.10 (p <0.01); Cd dose × Treatment: F – 46.83 (p <0.01)				
Pb				
Control (C)	57.130 ± 1.889 $^{c\,B}$	32.304 ± 1.339 $^{ef\,B}$	7.374 ± 0.811 $^{g\,B}$	32.269 ^{II}
Calcium carbonate (Ca)	62.745 ± 5.480 $^{bc\,B}$	14.028 ± 1.621 $^{g\,C}$	10.384 ± 1.167 $^{g\,B}$	29.053 ^{II}
Sepiolite (S)	42.255 ± 3.060 $^{d\,C}$	11.413 ± 1.010 $^{g\,C}$	11.608 ± 2.330 $^{g\,B}$	21.758 ^{III}
Expanded clay (E)	39.312 ± 3.460 $^{de\,C}$	27.650 ± 4.623 $^{f\,B}$	8.289 ± 0.833 $^{g\,B}$	25.084 ^{III}
Zeolite (Z)	73.151 ± 2.475 $^{a\,A}$	14.780 ± 2.336 $^{g\,C}$	7.124 ± 0.749 $^{g\,B}$	31.685 ^{II}
Molecular sieve (MS)	65.435 ± 2.202 $^{ab\,AB}$	43.300 ± 1.745 $^{d\,A}$	24.754 ± 4.001 $^{f\,A}$	44.496 ^{I}
Average	56.671 ^{Z}	23.912 ^{Y}	11.589 ^{X}	
Source of variation (F statistic and probability level):				
Cd dose: F – 1343.26 (p <0.01); Treatment: F – 76.21 (p <0.01); Cd dose × Treatment: F – 40.98 (p <0.01)				
Cr				
Control (C)	359.919 ± 18.014 $^{a\,A}$	98.464 ± 8.352 $^{ef\,B}$	29.942 ± 2374 $^{ij\,B}$	162.775 ^{II}
Calcium carbonate (Ca)	198.247 ± 8.605 $^{c\,C}$	55.420 ± 7.576 $^{ghi\,D}$	27.579 ± 3.601 $^{j\,B}$	93.749 ^{V}
Sepiolite (S)	188.192 ± 7.801 $^{cd\,C}$	39.497 ± 3.707 $^{hij\,E}$	31.582 ± 7.234 $^{ij\,B}$	86.424 ^{V}
Expanded clay (E)	211.296 ± 11.296 $^{c\,C}$	60.093 ± 7.124 $^{gh\,CD}$	30.712 ± 4.131 $^{ij\,B}$	100.700 ^{IV}
Zeolite (Z)	271.879 ± 7.242 $^{b\,B}$	73.790 ± 9.371 $^{fg\,C}$	30.797 ± 2.852 $^{ij\,B}$	125.489 ^{III}
Molecular sieve (MS)	297.620 ± 11.425 $^{b\,B}$	160.852 ± 4.615 $^{d\,A}$	107.036 ± 11.434 $^{e\,A}$	188.503 ^{I}
Average	254.526 ^{Z}	81.353 ^{Y}	42.941 ^{X}	
Source of variation (F statistic and probability level):				
Cd dose: F – 2784.80 (p <0.01); Treatment: F – 186.12 (p <0.01); Cd dose × Treatment: F – 42.93 (p <0.01)				
Cu				
Control (C)	178.423 ± 6.062 $^{b\,A}$	80.685 ± 4.107 $^{e\,B}$	41.038 ± 2.628 $^{g\,BC}$	100.049 ^{II}
Calcium carbonate (Ca)	137.454 ± 15.950 $^{cd\,B}$	50.666 ± 2.226 $^{fg\,CD}$	36.823 ± 4.628 $^{g\,C}$	74.981 ^{III}
Sepiolite (S)	121.158 ± 5.667 $^{d\,B}$	38.988 ± 4.289 $^{g\,D}$	42.414 ± 8.281 $^{g\,BC}$	67.520 ^{III}
Expanded clay (E)	180.359 ± 4.442 $^{b\,A}$	82.957 ± 11.634 $^{e\,B}$	56.539 ± 3.686 $^{fg\,B}$	106.618 ^{II}
Zeolite (Z)	197.129 ± 11.649 $^{b\,A}$	69.655 ± 3.554 $^{ef\,BC}$	36.160 ± 1.335 $^{g\,C}$	100.981 ^{II}
Molecular sieve (MS)	177.462 ± 6.885 $^{b\,A}$	235.511 ± 16.326 $^{a\,A}$	153.085 ± 16.326 $^{c\,A}$	188.686 ^{I}
Average	165.331 ^{Z}	93.077 ^{Y}	61.010 X	
Source of variation (F statistic and probability level):				
Cd dose: F – 897.00 (p <0.01); Treatment: F – 292.63 (p <0.01); Cd dose × Treatment: F – 65.53 (p <0.01)				

Zn				
Control (C)	1380.955 ± 47.795 $^{a\ A}$	744.505 ± 12.990 $^{ef\ B}$	332.908 ± 28.297 $^{h\ B}$	819.456 ^{II}
Calcium carbonate (Ca)	1021.325 ± 41.691 $^{d\ C}$	306.603 ± 21.700 $^{h\ C}$	224.332 ± 14.819 $^{hi\ C}$	517.420 ^{IV}
Sepiolite (S)	858.093 ± 25.257 $^{e\ D}$	168.018 ± 18.903 $^{I\ C}$	133.922 ± 25.090 $^{I\ D}$	386.677 ^{V}
Expanded clay (E)	1218.725 ± 40.459 $^{bc\ B}$	1039.533 ± 94.559 $^{d\ A}$	559.835 ± 28.265 $^{g\ A}$	939.364 ^{I}
Zeolite (Z)	1102.364 ± 17.431 $^{cd\ C}$	650.912 ± 25.382 $^{fg\ B}$	259.375 ± 8.965 $^{hi\ BC}$	670.884 ^{III}
Molecular sieve (MS)	1276.029 ± 47.204 $^{ab\ B}$	793.086 ± 36.198 $^{e\ B}$	593.031 ± 75.140 $^{g\ A}$	887.382 ^{I}
Average	1142.915 ^{Z}	617.109 ^{Y}	350.567 ^{X}	
Source of variation (F statistic and probability level):				
Cd dose: F – 1610.62 ($p<0.01$); Treatment: F – 236.74 ($p<0.01$); Cd dose × Treatment: F – 26.39 ($p<0.01$)				
Ni				
Control (C)	236.217 ± 10.894 $^{a\ A}$	110.901 ± 0.752 $^{ef\ A}$	38.299 ± 2.737 $^{h\ BC}$	128.472 ^{I}
Calcium carbonate (Ca)	122.567 ± 5.215 $^{de\ D}$	43.301 ± 1.906 $^{h\ E}$	31.211 ± 2.474 $^{h\ C}$	65.693 ^{V}
Sepiolite (S)	117.155 ± 3.792 $^{de\ D}$	35.201 ± 2.252 $^{h\ F}$	34.038 ± 6.104 $^{h\ BC}$	62.131 ^{V}
Expanded clay (E)	144.226 ± 13.340 $^{c\ C}$	67.271 ± 4.344 $^{g\ D}$	43.879 ± 2.809 $^{h\ B}$	85.125 ^{IV}
Zeolite (Z)	167.626 ± 2.981 $^{b\ B}$	79.864 ± 7.329 $^{g\ C}$	36.148 ± 3.490 $^{h\ BC}$	94.546 ^{III}
Molecular sieve (MS)	130.746 ± 3.159 $^{cd\ CD}$	99.649 ± 4.549 $^{fg\ B}$	81.446 ± 8.335 $^{g\ A}$	103.947 ^{II}
Average	153.090 ^{Z}	72.698 ^{Y}	44.170 ^{Z}	
Source of variation (F statistic and probability level):				
Cd dose: F – 2296.13 ($p<0.01$); Treatment: F – 221.49 ($p<0.01$); Cd dose × Treatment: F – 83.31 ($p<0.01$)				
Fe				
Control (C)	33,651.593 ± 1555.263 $^{b\ B}$	10,170.735 ± 257.548 $^{de\ C}$	4783.155 ± 361.386 $^{g\ B}$	16201.827 ^{III}
Calcium carbonate (Ca)	33,776.728 ± 2999.066 $^{b\ AB}$	6570.564 ± 367.751 $^{fg\ D}$	3589.622 ± 181.227 $^{g\ B}$	14645.638 ^{III}
Sepiolite (S)	33,770.761 ± 2333.610 $^{b\ AB}$	5350.771 ± 276.832 $^{g\ D}$	5346.763 ± 1054.401 $^{g\ B}$	14822.765 ^{III}
Expanded clay (E)	33,286.210 ± 928.092 $^{b\ B}$	9101.578 ± 414.724 $^{ef\ C}$	5304.643 ± 485.619 $^{g\ B}$	15897.477 ^{III}
Zeolite (Z)	38231.428 ± 1085.522 $^{a\ A}$	12,967.423 ± 1171.848 $^{cd\ B}$	5520.882 ± 561.291 $^{g\ B}$	18906.578 ^{II}
Molecular sieve (MS)	33,436.284 ± 1554.340 $^{b\ B}$	15,551.628 ± 994.458 $^{c\ A}$	12,839.676 ± 1292.640 $^{cd\ A}$	20609.196 ^{I}
Average	34,358.834 ^{Z}	9952.116 ^{Y}	6230.790 ^{X}	
Source of variation (F statistic and probability level):				
Cd dose: F – 3061.56 ($p<0.01$); Treatment: F – 37.61 ($p<0.01$); Cd dose × Treatment: F – 13.35 ($p<0.01$)				
Mn				
Control (C)	4988.153 ± 148.305 $^{a\ A}$	3216.047 ± 17.864 $^{e\ B}$	1240.502 ± 115.143 $^{g\ BC}$	3148.234 ^{II}
Calcium carbonate (Ca)	3846.912 ± 159.747 $^{d\ C}$	1391.814 ± 104.200 $^{g\ C}$	1086.105 ± 34.789 $^{gh\ C}$	2108.277 ^{IV}
Sepiolite (S)	3666.757 ± 121.350 $^{d\ C}$	1100.952 ± 99.501 $^{gh\ C}$	1052.966 ± 234.424 $^{gh\ CD}$	1940.225 ^{IV}
Expanded clay (E)	4335.343 ± 112.390 $^{bc\ B}$	3920.506 ± 519.687 $^{cd\ A}$	2189.825 ± 113.711 $^{f\ A}$	3481.891 ^{I}
Zeolite (Z)	4387.338 ± 92.468 $^{b\ B}$	3012.902 ± 123.322 $^{e\ B}$	1441.300 ± 54.301 $^{g\ B}$	2947.180 ^{III}
Molecular sieve (MS)	1929.813 ± 79.313 $^{f\ D}$	1230.102 ± 34.093 $^{g\ C}$	788.354 ± 97.381 $^{h\ D}$	1316.090 ^{V}
Average	3859.053 ^{Z}	2312.054 ^{Y}	1299.842 ^{X}	
Source of variation (F statistic and probability level):				
Cd dose: F – 1458.78 ($p<0.01$); Treatment: F – 303.02 ($p<0.01$); Cd dose × Treatment: F – 54.85 ($p<0.01$)				

Lowercase letters indicate significant differences for both variables, i.e., Cd dose and mineral amendments; uppercase letters indicate significant differences among sorbent types separately for each Cd dose; Roman numerals indicate significant differences among mean values for mineral amendments, irrespective of Cd dose; and the letters X, Y, and Z indicate significant differences among mean values for Cd doses, irrespective of mineral amendments.
